# IFNγ is a central node of cancer immune equilibrium

**DOI:** 10.1016/j.celrep.2023.112219

**Published:** 2023-03-06

**Authors:** Michael J. Walsh, Courtney T. Stump, Rakeeb Kureshi, Patrick Lenehan, Lestat R. Ali, Michael Dougan, David M. Knipe, Stephanie K. Dougan

**Affiliations:** 1 Department of Microbiology, Blavatnik Institute, Harvard Medical School, Boston, MA 02115, USA; 2 Harvard Program in Virology, Boston, MA, USA; 3 Department of Cancer Immunology and Virology, Dana-Farber Cancer Institute, Boston, MA 02215, USA; 4 Division of Gastroenterology, Department of Medicine, Massachusetts General Hospital, Boston, MA 02114, USA; 5 Department of Immunology, Harvard Medical School, Boston, MA, USA; 6 Department of Medicine, Harvard Medical School, Boston, MA, USA; 7 These authors contributed equally; 8 Lead contact

## Abstract

Tumors in immune equilibrium are held in balance between outgrowth and destruction by the immune system. The equilibrium phase defines the duration of clinical remission and stable disease, and escape from equilibrium remains a major clinical problem. Using a non-replicating HSV-1 vector expressing interleukin-12 (*d*106S-IL12), we developed a mouse model of therapy-induced immune equilibrium, a phenomenon previously seen only in humans. This immune equilibrium was centrally reliant on interferon-γ (IFNγ). CD8^+^ T cell direct recognition of MHC class I, perforin/granzyme-mediated cytotoxicity, and extrinsic death receptor signaling such as Fas/FasL were all individually dispensable for equilibrium. IFNγ was critically important and played redundant roles in host and tumor cells such that IFNγ sensing in either compartment was sufficient for immune equilibrium. We propose that these redundant mechanisms of action are integrated by IFNγ to protect from oncogenic or chronic viral threats and establish IFNγ as a central node in therapy-induced immune equilibrium.

## In brief

Walsh et al. develop a mouse model of therapy-induced cancer immune equilibrium through injection of a non-replicating interleukin-12-expressing virus. Although interferon-γ (IFNγ) was essential for establishment and maintenance of equilibrium, tumor or host sensing of IFNγ was redundant and tumors were controlled without direct T cell cytotoxicity.

## Graphical Abstract

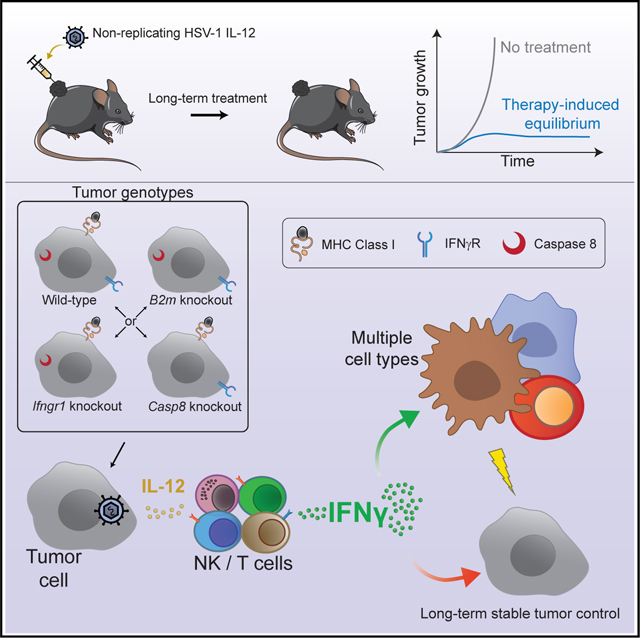

## INTRODUCTION

Immunotherapies harness the ability of the immune system to recognize and destroy cancer cells. Cytokine-based therapies, adoptive cell therapies, and antibody blockade of negative regulatory pathways such as cytotoxic T lymphocyte-associated protein 4 (CTLA-4) and programmed death (PD)-1/L1 have demonstrated the effectiveness of immune-stimulating agents for cancer treatment.^1^ In both humans and mice, the immune system also surveys for nascent cancer development, although the extent to which immune surveillance happens in the absence of therapy is difficult to quantify. Immune equilibrium, or a state of balance between the immune system and clinically undetected or stable tumors, occurs as demonstrated by elegantly designed mouse models and the observation of very long latency periods for certain cancer types such as melanoma.^2,3^ Many immunotherapies can induce an equilibrium-like state in patients with tumors held in check until immune escape promotes tumor progression.^4^ Among 105 melanoma patients from the KEYNOTE-001 trial initially classified as having a complete response following anti-PD1 treatment, long-term follow-up revealed that 12% of these complete responders eventually had detectable disease, suggesting their tumors had been held in clinically undetectable equilibrium.^5^ This equilibrium state in patients is poorly understood and remains a significant clinical barrier to successfully curing patients.

Interferon-γ (IFNγ) signaling is important for the initial response to checkpoint blockade, as highlighted by several CRISPR screens^6–8^ and evaluation of patients receiving CTLA-4 blockade who displayed primary or acquired resistance.^9,10^ These studies have found various mutations that inactivate IFNγ sensing in the tumor cells and tend to promote resistance to immunotherapy. In this early response, IFNγ is thought to slow tumor growth and improve CD8^+^ T cell-directed responses via enhanced antigen presentation.^11^ IFNγ can also drive regulatory T cell (Treg) fragility in which Foxp3^+^ Tregs become less suppressive and can secrete IFNγ themselves, allowing for better antitumor responses.^12^ Additionally, IFNγ can act on the tumor stromal cells, inducing regression of the vasculature supporting tumors, ultimately causing tumor shrinkage.^13,14^ However, the long-term role of IFNγ in immune equilibrium is unclear. Biopsies cannot be obtained from patients with clinically undetectable disease, and we lack a mouse model of therapy-induced equilibrium. IFNγ has several negative feedback mechanisms, and chronic IFNγ signaling can promote tumor outgrowth.^15,16^ It is therefore unknown whether long-term exposure to IFNγ is beneficial or deleterious for tumor control.

Viruses can provide an alternative method of immunotherapy. Viral nucleic acids or glycoproteins can serve as pathogen-associated molecular patterns that engage Toll-like receptors (TLRs), such as TLR2 and TLR9,^17–19^ or other pattern recognition receptors (PRRs),^20^ to generate an inflammatory response and stimulate maturation of dendritic cells (DCs) to efficiently prime a T cell response against the tumor. Recent advances in genetic engineering of viruses have improved their therapeutic potential, and in 2015 the Food and Drug Administration approved the use of the first oncolytic virus, talimogene laherparepvec (T-VEC), a replication-competent herpes simplex virus 1 (HSV-1) recombinant encoding granulocyte-macrophage colony-stimulating factor (GM-CSF), for the local treatment of melanoma metastases.^21^

Interleukin-12 (IL-12) is a potent cytokine with multiple functions, including the abilities to stimulate IFNγ production and enhance the growth and cytotoxicity of natural killer (NK), CD8, and CD4 T cells.^22^ IL-12 also has antiangiogenic properties. Methods of introducing IL-12 into the tumor microenvironment have included recombinant protein injection,^23,24^ plasmid electroporation,^25,26^ and microspheres,^27^ as well as oncolytic virus delivery through both replication-competent^28–34^ and defective^35,36^ viral vectors. However, owing to the pleiotropic effects of IL-12, dose-limiting toxicities are a substantial barrier to effective treatment in patients.^37^ In addition, the standard oncolytic viruses may generate illness in some patients and cannot be given to immunosuppressed patients because they are replication competent, with toxicity increasing further when used as part of a combination therapy.^38–40^

To improve the safety profile of such oncolytic viruses, we took advantage of the HSV-1 *d*106S vaccine vector, which unlike T-VEC and most other oncolytic viruses is replication defective.^41–43^ This virus expresses a GFP viral transgene, which can be replaced with any protein of interest, such as a tumor-specific antigen,^44^ therapeutic checkpoint inhibitor antibody, or cytokine. We chose to use *d*106S as a vector to induce a large burst of IL-12 locally within the tumor environment. We discovered that local delivery of IL-12 with the *d*106S-IL12 vector led to long-term stable equilibrium of B16 melanoma tumors, lasting over 120 days whereby the mice neither clear nor succumb to their tumors. We identify IFNγ as the central factor required for establishment and maintenance of therapy-induced equilibrium and demonstrate redundant mechanisms of action for this critical cytokine.

## RESULTS

### *d*106S-IL12 is a model for therapeutic induction of immune equilibrium

Although human cells are readily infectable by HSV-1, B16 murine melanoma cells are non-permissive due to lack of necessary entry receptors (Figure S1A). We therefore transduced B16 melanoma cells with a lentivirus encoding human nectin-1,^45^ a known HSV-1 entry receptor.^46^ Whereas B16^WT^ cells could not be infected with *d*106S-GFP, B16^Nectin1^ cells were readily infected with the virus; titration of *d*106S-GFP virus on B16^Nectin1^ alongside a panel of five human melanoma cell lines showed comparable levels of infection (Figure S1B). Thus, B16^Nectin1^ cells faithfully model *d*106S entry into human melanoma cells.

We chose IL-12 as a cargo for local virotherapy given the known role of this cytokine in orchestrating T helper 1 (Th1) responses and the limitations associated with its systemic delivery. We inserted an IL-12 fusion gene cassette in place of GFP to generate the *d*106S-IL12 virus (Figure S1C) and measured IL-12 (p40) concentration in infected tumor cell supernatants by ELISA. Even at the lowest MOI tested, 0.1 plaque-forming units (PFU)/cell, *d*106S-IL12 produced abundant levels of cytokine: 9,000 pg per 10^5^ cells over 24 h (Figure S1D). We tested whether *d*106S-IL12 could robustly induce IL-12 secretion *in vivo* by inoculating mice with a subcutaneous B16^Nectin1^ tumor and injecting intratumorally with PBS, *d*106S, or *d*106S-IL12 every 3 days starting at day 7. Tumors were collected at day 10 or day 17 post challenge and showed robust levels of IL-12 present (Figure S1E). Our HSV-1 *d*106S expresses the single-chain version of IL-12, allowing for robust and stable secretion of both subunits, without antagonism from the p40 subunit expression alone.^47–49^ In addition, the levels of IL-12 secretion that we achieved with *d*106S-IL12 infection were higher than with previously reported IL-12-expressing oncolytic viruses,^28–30,35^ demonstrating the robust gene expression capabilities of this vector.

We expected that the viral vector itself would induce an IFN response,^50^ although this response could be dampened through expression of the viral ICP0 protein.^51,52^ To examine this, we infected B16^Nectin1^ cells with *d*106S or *d*106S-IL12 virus and co-cultured these cells with murine bone marrow dendritic cells (BMDCs), or separately stimulated BMDCs with virus alone. We measured mRNA expression with a panel of inflammatory genes including IFN-stimulated genes (ISGs) and inflammatory nuclear factor-κB genes. As previously shown in other cell types,^51,52^ the *d*106S infection resulted in a minor type I IFN response within the melanoma cells, as shown by IFN-β(*IFNb1*) expression (Figure S1F). However, co-culture of infected B16 with DCs resulted in a more robust IFN response from DCs (Figures S1F–S1H), which are themselves non-permissive to infection^53^ but can robustly induce IFN production via innate sensors.

**Figure 1.**
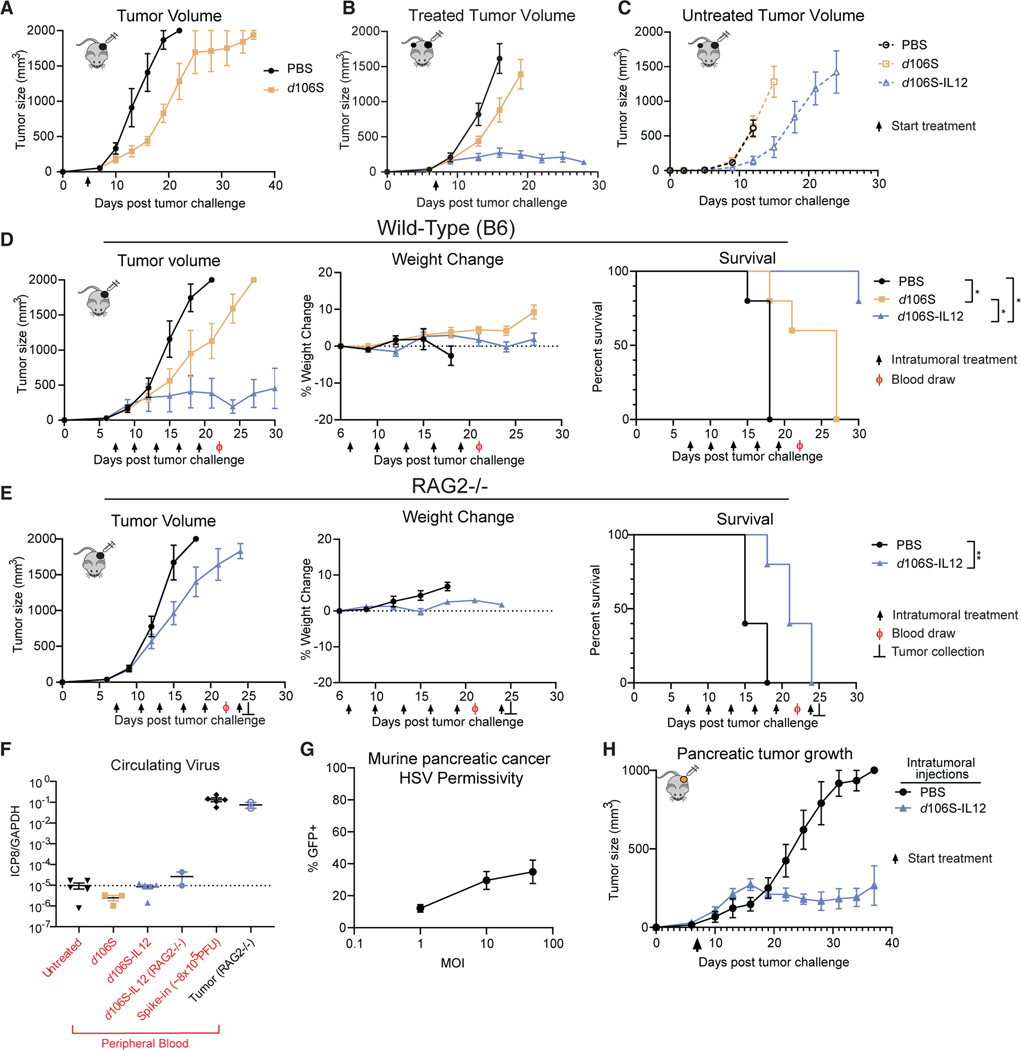
A mouse model of therapy-induced immune equilibrium (A) C57BL/6J mice were challenged with 5 × 10^5^ B16^Nectin1^ cells subcutaneously in the flank. Following 5 days of tumor growth, mice were randomized into treatment groups (N = 6 per group) and received intratumoral injection of 30 μL of either PBS or *d*106S (1.5 × 10^7^ PFU) every 3 days. (B and C) Mice were challenged with a primary B16^Nectin1^ tumor (N = 10 per group) followed by a secondary, contralateral tumor 4 days later. Seven days after initial challenge, only the primary tumor was injected every 3 days with PBS, *d*106S, or *d*106S-IL12 for a total of 5 injections. (D and E) Wild-type C57BL/6J (D) or RAG2^−/−^ mice (E) were inoculated with unilateral B16^Nectin1^ tumors and injected intratumorally every 3 days with PBS, *d*106S (wild-type mice only), or *d*106S-IL12 for a total of 5 injections (N = 5 per group) starting on day 7 for a total of 5 injections. Weight change was normalized to immediately before treatment began. Arrows indicate treatment; Ф indicates blood draw; ⊥ indicates RAG2^−/−^ sacrifice (2 mice) for tumor DNA extraction. (F) qPCR for viral DNA (ICP8) from DNA extracted from peripheral blood draw (day 22 surviving mice) and tumor (day 25 RAG2^−/−^). Untreated mice also bore tumors but did not receive treatment. Spike-in control received 8 × 10^5^ PFU of virus into untreated blood. Dotted line indicates limit of detection based on untreated samples. (G) Wild-type 6694c2 pancreatic cancer cells were infected with varying MOIs of *d*106S (GFP) and infection measured by flow cytometry (n = 4). (H) Mice were challenged subcutaneously with 2 × 10^5^ 6694c2 cells and treated every 3 days starting on day 7 (N = 5 PBS, N = 5 *d*106S-IL12). Values are mean ± SEM. Survival groups were compared using a Bonferroni-corrected log-rank test. *p < 0.05; **p < 0.01.

To examine whether the *in vitro* inflammatory response induced by *d*106S had an *in vivo* effect on tumor growth, we challenged mice with B16^Nectin1^ tumors by subcutaneous injection into the flank. After 5 days of tumor growth, mice were randomized and injected intratumorally with either PBS or *d*106S (GFP) every 3 days, and tumor growth and survival were monitored. The injection of *d*106S virus slowed tumor growth (Figure 1A), indicating that some aspects of the viral vector, including the IFN response, were sufficient to delay tumor growth, albeit modestly.

To test the efficacy of *d*106S-IL12 therapy, we inoculated mice with a primary tumor and 4 days later inoculated a secondary tumor on the contralateral flank. One week after primary tumor challenge, mice were injected with either PBS, *d*106S, or *d*106S-IL12 intratumorally into only the primary tumor. Growth of the primary, treated tumor was reduced slightly with *d*106S, as seen previously (Figure 1B) and reduced more with *d*106S-IL12 (Figure 1B). However, *d*106S failed to reduce growth of the secondary, untreated tumor, while *d*106S-IL12 partially reduced growth of the distal tumor (Figure 1C), suggesting that *d*106S acted locally while IL-12 induced a systemic immune response.

Not only did the tumors directly injected with *d*106S-IL12 have reduced growth compared with *d*106S but their size remained relatively fixed after several doses, suggesting establishment of equilibrium. However, because the response of contralateral, uninjected tumors was insufficient to stably control tumors, we wished to test whether this equilibrium was due to innate immune activation at the site of injection or induction of adaptive immunity. We inoculated wild-type C57BL/6 and RAG2^−/−^ immunodeficient mice with tumors. In wild-type mice, *d*106S-IL12 again led to a slowing of tumor growth and prolonged survival with stable tumor masses (Figure 1D). However, in RAG2^−/−^ mice, *d*106S-IL12 led to only a partial slowing of tumor growth and no equilibrium (Figure 1E), suggesting that adaptive immunity was necessary to stably control tumors. Weight loss was monitored as a proxy for toxicity, as systemic IL-12 in humans and mice is limited by severe toxicity;^24,37^ no major weight loss was seen in either wild-type or RAG2^−/−^ mice (Figures 1D and 1E). All remaining mice were bled at day 22; DNA was extracted from the peripheral blood and from RAG2^−/−^ tumors at 24 h post viral injection to measure levels of circulating free virus by qPCR for the viral gene *ICP8* gene. The level of viral DNA in the blood was at undetectable levels compared with the levels found in the tumor (Figure 1F). In total, these results showed a focused local infection, non-toxicity of the *d*106S vector even in immunocompromised hosts, and the necessity for adaptive immunity to induce equilibrium in response to *d*106S-IL12.

We then tested whether equilibrium could be established in another model using the pancreatic cancer clone 6694c2,^54^ which is also known to be resistant to checkpoint blockade.^54,55^ Without genetic perturbation, these 6694c2 cells were naturally permissive to HSV infection, unlike native B16 (Figure S1A), although less well infected than B16^Nectin1^ (Figures 1G and S1B). Even with lower levels of HSV infection, *d*106S-IL12 could readily control cc6942 tumors and ultimately establish equilibrium (Figure 1H). This indicated that equilibrium establishment using *d*106S-IL12 could occur in multiple murine tumor models.

### Tumor-intrinsic resistance does not facilitate outgrowth in response to *d*106S-IL12

**Figure 2.**
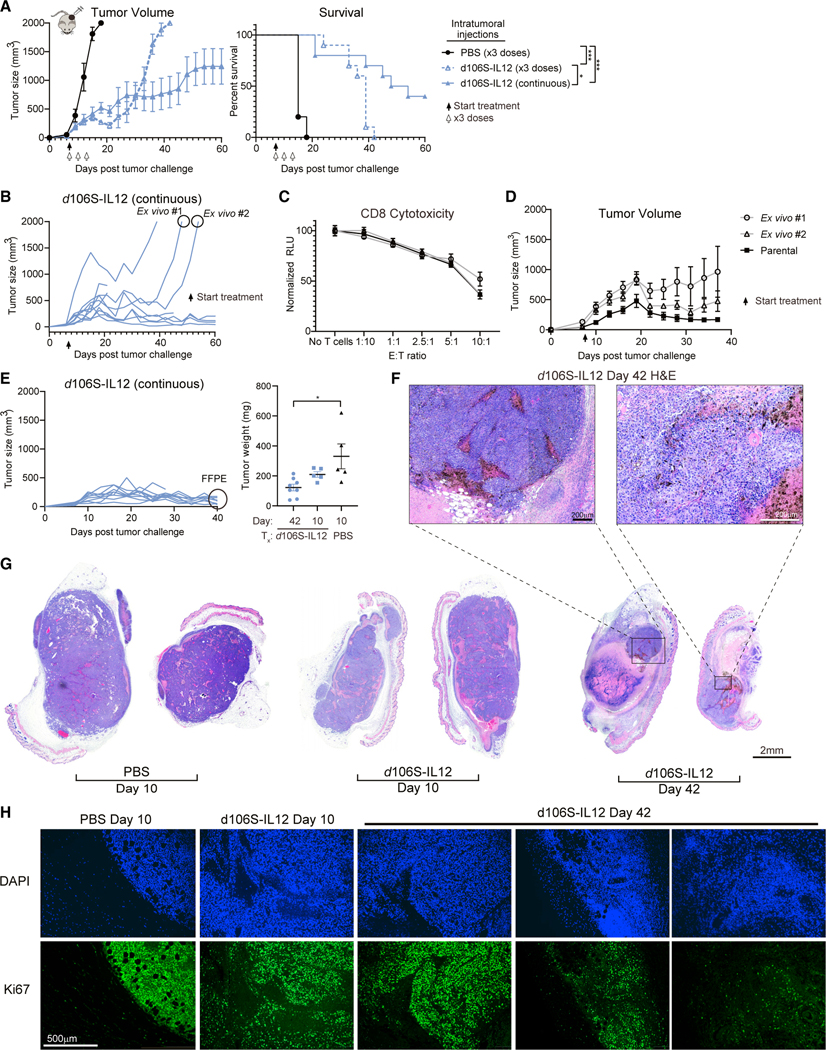
Ongoing treatment with *d*106S-IL12 generates immune equilibrium but not intrinsic resistance (A) Mice were challenged with unilateral B16^Nectin1^ tumors (5 × 10^5^ cells) and received either continuous intratumoral dosing every 3 days or only 3 total doses as indicated starting at day 7 (N = 5 mice for PBS, N = 10 mice per *d*106S-IL12 group). Solid arrow indicates start of continuous dosing; white arrows indicate 3 doses. (B) Individual growth curves from (A); 2 outgrowing tumors were collected for *ex vivo* culture. (C) Activated CD8^+^ TRP1^high^ cells were co-cultured for 24 h with IFNγ-stimulated *ex vivo* or parental B16^Nectin1^ cells, and cytotoxicity was measured by CellTiter-Glo. See also Figure S2. Effector/target (E:T) ratios are shown. (D) Mice were challenged with 5 × 10^5^ cells from either *ex vivo* cultures or B16^Nectin1^ parental line (N = 5 per tumor type). Continuous treatment with *d*106SIL12 began at day 7, as indicated by the arrow. (E) B16^Nectin1^ tumors were treated continuously with *d*106S-IL12 as before and collected at day 42 post tumor growth (N = 8) or separately treated once with PBS or *d*106S-IL12 and collected at day 10 (N = 5). Tumor weights are shown. Tumors were formalin fixed and paraffin embedded (FFPE) and stained with hematoxylin and eosin (H&E). (F and G) Representative H&E of whole tumor slices (G) or zoomed in on day 42 tumors (F). (H) FFPE sections were also stained with anti-Ki67 and DAPI and imaged by immunofluorescence. See also Figure S3. Values are mean ± SEM. Tumor weights were compared with a one-way ANOVA and Tukey’s test; survival was compared using a Bonferroni-corrected log-rank test. *p < 0.05.

To better understand the mechanism of equilibrium, we wanted to determine the requirement for ongoing treatment. We tested whether three total doses or continuous treatment of mice every 3 days could induce stable equilibrium. Despite an initial reduction in B16 tumor size with only three treatments, all mice with treatment cessation failed to clear their tumors; their tumors grew out and they did not survive for as long as mice receiving continuous *d*106S-IL12 therapy (Figure 2A). However, even with continuous dosing of mice, all mice failed to fully clear their tumors and, instead, the majority entered an equilibrium phase (Figures 2A and 2B). Therefore, we concluded that continuous therapy was necessary to keep tumors in immune equilibrium. However, immune equilibrium was not established in all tumors, as some did not appear to respond to therapy and continued to grow out during the treatment window (Figure 2B), suggesting a means of resistance.

To determine whether resistance was due to mutations in the tumor permitting immune escape or other mechanisms, we cultured two of these outgrowing tumors *ex vivo* to generate cell lines (Figure 2B). One possible mechanism of resistance could be loss of nectin-1, the HSV entry receptor, therefore preventing viral transduction and IL-12 production. We determined by flow cytometry that nectin-1 levels were modestly reduced, resulting in a 5%–50% reduction in infectivity (Figures S2A and S2B). However, given the high expression of IL-12 from this vector (Figures S1C and S1D), this reduction in infectivity is unlikely to fully explain the rapid outgrowth of the tumor, so we investigated alternative mechanisms of resistance.

Another possible mechanism of resistance was reduction in major histocompatibility complex (MHC) class I levels, which allows tumors cells to avoid immune surveillance by CD8^+^ T cells.^56^ However, we found that the *ex vivo* tumor cells had higher levels of MHC class I on their surface compared with the parental tumor cell line, in both the absence and presence of IFNγ stimulation (Figure S2C). Additionally, levels of PD-L1 were not enhanced on the surface of these cells (Figure S2D). Co-culture of *ex vivo* tumor cells with melanocyte-specific TRP1^high^ effector CD8^+^ T cells^57^ showed no resistance to T cell cytotoxicity compared with the parental tumor cell line (Figure 2C). These results suggested that the tumor outgrowth seen *in vivo* was likely tumor cell extrinsic. To test this, we challenged mice with either the parental tumor cell line or the *ex vivo* tumor cells and began continuous injection with *d*106S-IL12 virus on day 7. Although the *ex vivo* lines grew slightly faster, treatment with *d*106S-IL12 still had a therapeutic benefit, as all tumors responded to therapy (Figure 2D). We therefore conclude that their original rapid outgrowth was due to stochastic loss of immune equilibrium rather than tumor-intrinsic factors.

### Cellular characteristics of tumors treated with IL-12 virotherapy

To confirm that continuously treated tumors were indeed live cells held in equilibrium rather than a mass of dead cells, we collected day-42 tumors from mice dosed every 3 days with IL-12 virotherapy and day-10 tumors from mice treated with PBS or *d*106S-IL12 only once (Figure 2E). From whole sections of this tissue, the tumor was readily identifiable even at day 42, although there was an increase in the portion of necrotic tissue compared with day-10 PBS or *d*106S-IL12 tumors (Figures 2F and 2G). Ki67 staining revealed a heterogeneous mix of high, medium, and low areas of tumor proliferation at day 42 (Figure 2H). These results indicated that the tumors held in therapy-induced equilibrium were indeed viable and actively proliferating.

These paraffin-embedded tumors were stained for cleaved caspase 3 or Ki67 by immunohistochemistry (Figure S3A), and the ratio of Ki67 to cleaved caspase 3 was determined. There was a significantly higher ratio of Ki67 to cleaved caspase 3 in day-10 PBS tumors compared with day-42 *d*106S-IL12 tumors, suggesting higher levels of proliferation in these control-treated tumors (Figure S3B). Interestingly, by day 42 this ratio of Ki67 to cleaved caspase 3 had reached 1, suggesting tumors were in equilibrium (Figure S3B). Tumors were also collected from mice on day 17 and stained by immunohistochemistry for CD31 to image the tumor vasculature. We saw no decrease in the quantity of CD31 staining in tumors treated with *d*106S-IL12 (Figures S3C and S3D).

**Figure 3.**
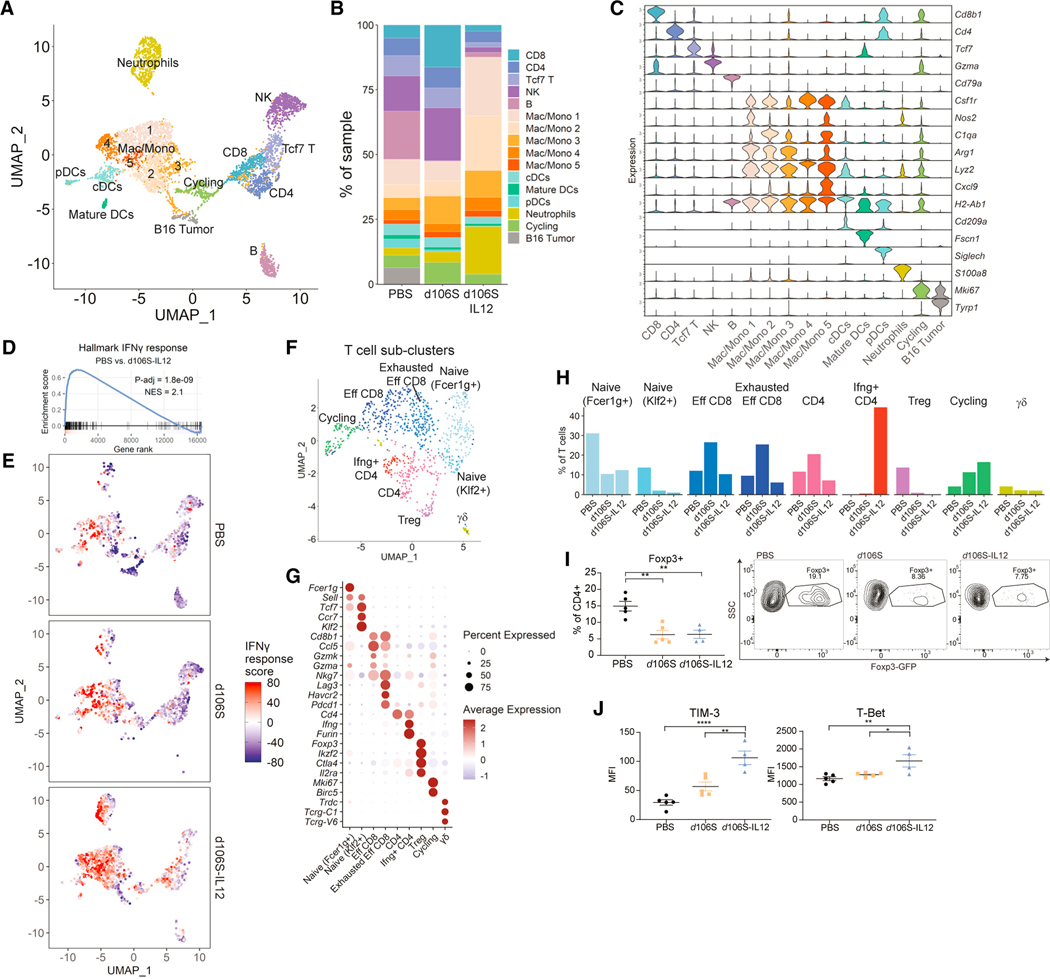
Single-cell RNA sequencing reveals IFNγ response with d106S-IL12 and reduction in Tregs Mice with B16^Nectin1^ tumors were treated 4 times in total and sacrificed on day 16 (PBS) or day 17 (*d*106S/*d*106S-IL12). Tumors from each group were pooled (n = 5) and sorted for CD45^+^ live cells and subjected to single-cell RNA sequencing. (A) Unsupervised clustering analysis and resulting uniform manifold approximation and projection (UMAP) of all cells. (B and C) Cell cluster frequency (B) and violin plot (C) of canonical cluster marker genes. (D) GSEA for hallmark IFNγ-response genes. (E) IFNγ-response score UMAP based on scaled and summed expression of hallmark IFNγ-response genes for each cell. (F and G) Subclustering of T cells revealed several unique clusters (F) based on canonical gene expression (G). (H) Distribution of T cell subclusters across treatment conditions. See also Figure S4. (I) Foxp3-GFP mice were challenged with B16^Nectin1^ tumors and injected 4 times every 3 days with PBS, *d*106S, or *d*106S-IL12 (N = 5 PBS/*d*106S, N = 4 *d*106S-IL12) starting on day 7 post-tumor challenge. On day 17, tumors were collected for flow cytometry and analyzed for presence of Foxp3-GFP^+^ CD4^+^ T cells. Representative flow plots are shown. (J) Extracellular staining for TIM-3 and intracellular staining for T bet was performed on mice from (I). Groups were compared with one-way ANOVA and Tukey’s multiple comparisons test. NES, normalized enrichment score; MFI, mean fluorescence intensity. *p < 0.05; **p < 0.01; ****p < 0.0001.

To profile the immune infiltrate in these tumors treated with IL-12 virotherapy, we analyzed treated tumors by flow cytometry and single-cell RNA sequencing (scRNA-seq). We prepared scRNA-seq libraries from CD45^+^ live cells and identified 16 distinct clusters (Figure 3A) based on expression of various genes (Figure 3C). We saw differences in immune cell populations (Figure 3B) that were recapitulated by flow cytometry of these same tumors (Figures S4A and S4B). *d*106S-IL12 induced an early increase in CD8^+^ T cell infiltration at day 10, which returned to near baseline by day 17, coinciding with an increase in granulocytes and monocytic cells (Figures 3B, S4A, and S4B). We performed gene set enrichment analysis (GSEA) to compare PBS-treated tumors with *d*106S-IL12-treated tumors. One of the top pathways upregulated by IL-12 virotherapy was IFNγ response (Figure 3D). IFNγ-response genes were most prominently expressed in macrophage/monocyte and neutrophil clusters, although there was a general increase in IFNγ-response genes from all cell types (Figure 3E).

Subclustering of the T cell scRNA-seq data further delineated T cell subsets (Figure 3F) which were identified by canonical marker expression (Figure 3G). We discovered an increase in CD4^+^ T cells cells expressing high levels of IFNγ present in samples treated with *d*106S-IL12 (Figure 3H). There was also a marked absence of Foxp3^+^ Tregs in both samples treated with virus (Figure 3H). To confirm these findings, we inoculated Foxp3-GFP mice with B16^Nectin1^ tumors and treated them as in our scRNA-seq experiment. Flow cytometry revealed a similar decrease in Tregs in both *d*106S- and *d*106S-IL12-treated tumors (Figure 3I). Additionally, we measured TIM-3 expression, which is regulated by the Th1 transcriptional regulator, T-bet, and can serve as a marker of Th1 cells.^58^ We saw an increase in both TIM-3 and T-bet staining by flow cytometry of CD4^+^ T cells (Figure 3J), consistent with an expansion of Th1 cells.^59^

### IFNγ is a critical node for establishing and maintaining immune equilibrium

**Figure 4.**
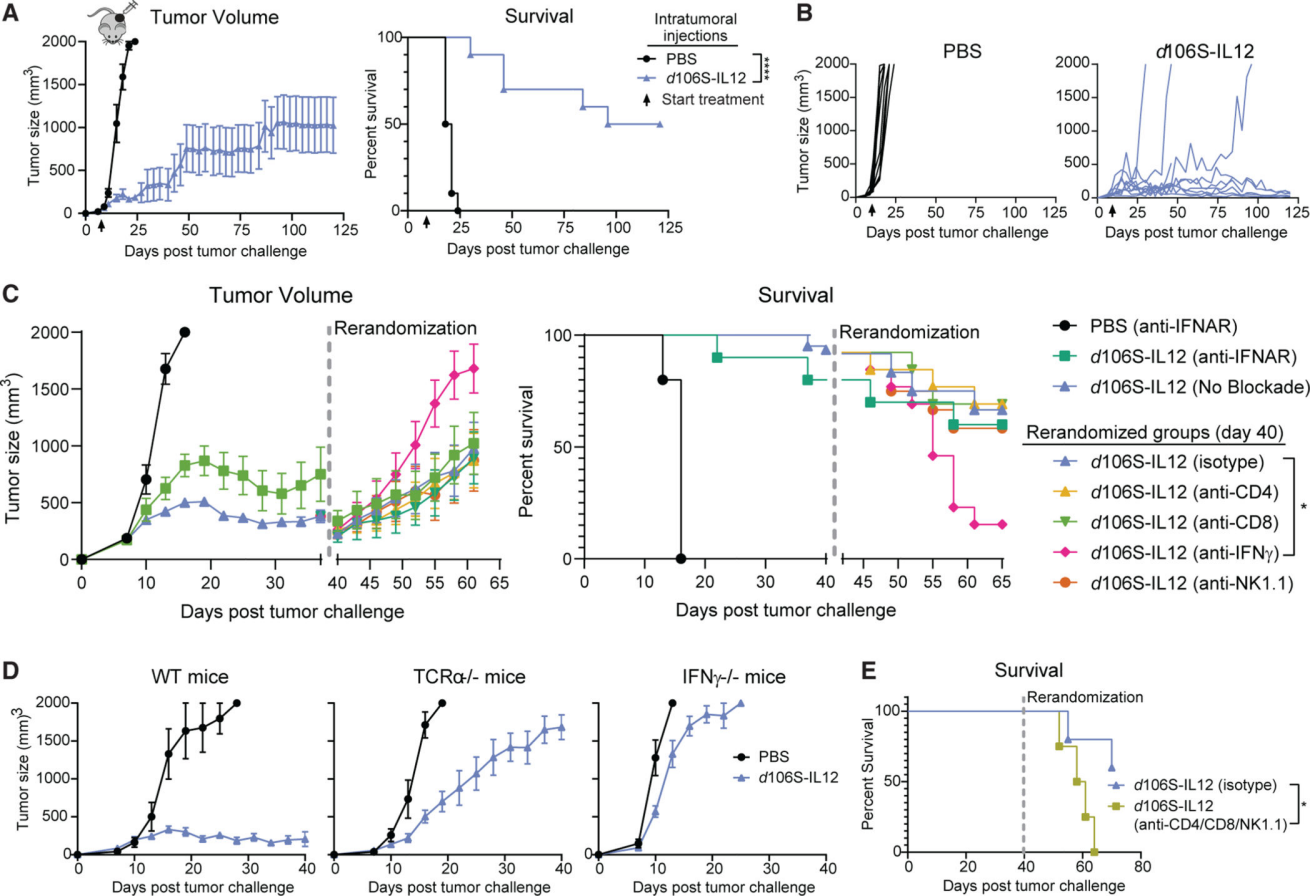
Continuous *d*106S-IL12 injections lead to stable tumor control dependent on IFNγ (A) Mice treated continuously starting at day 7 (arrow) (N = 5 for PBS; N = 10 for *d*106S-IL12). (B) Individual tumor growth from (A). (C) Mice with B16 tumors began intratumoral treatment with PBS or *d*106S-IL12 on day 7 and proceeded with treatment every 3 days. Some mice were also treated intraperitoneally (i.p.) with anti-TNFΑR blocking antibodies (100 mg) every 3 days (N = 5 for PBS, N = 10 for *d*106S-IL12), while the rest of the mice received no blockade (N = 62). At day 40, surviving mice were rerandomized to new treatment groups and injected i.p. with either isotype control, anti-CD4, anti-CD8, anti-IFNγ, or anti-NK1.1 depleting antibodies (100 mg) every 3 days while also continuing to receive *d*106S-IL12 treatment (curves go down in size as the rerandomized groups contain only remaining live mice, while left-side curves account for endpoint tumors). See also Figure S5. (D) Wild-type, TCRα^−/−^, or IFNγ^−/−^ mice (N = 5/10, 5/8, 5/10 mice for PBS/*d*106S-IL12) were treated with *d*106S-IL12 or PBS every 3 days. (E) Wild-type mice were treated as in (C) and rerandomized at day 40 into isotype control (N = 5) or anti-CD4^+^ anti-CD8^+^ anti-NK1.1 (100 μg each; N = 4) treatment every 3 days. Values are mean ± SEM. Survival was compared using a Bonferroni-corrected log-rank test. *p < 0.05.

Next, we wanted to study the cellular and molecular determinants for establishment and maintenance of equilibrium. Long-term experiments with B16^Nectin1^ confirmed that continuous dosing of mice with *d*106S-IL12 every 3 days led to reductions in tumor size and long-term sustained equilibrium, maintaining stable masses for more than 100 days (Figures 4A and 4B). Previous work has implicated IFNγ and T cells as controlling tumors in spontaneous equilibrium,^3^ although it is unclear what role they play in therapy-induced equilibrium. Because the *d*106S vector could induce a type I IFN response, we first tested whether blocking this response early in treatment with anti-IFNα/β receptor (TNFΑR) affected establishment of equilibrium. Anti-TNFΑR antibodies were given every 3 days starting with the first dose of *d*106S-IL12. TNFΑR blockade caused a more rapid initial growth of tumors treated with *d*106S-IL12, but mice treated with blocking antibodies ultimately stabilized their tumors around days 20–22 and were controlled, though at a higher overall set point than mice not receiving TNFΑR blockade (Figure 4C, left). This indicates that the establishment of equilibrium can occur at a range of tumor sizes and does not necessitate type I IFN.

We then tested whether perturbing the equilibrium state would allow for tumor outgrowth, as this was the key parameter initially used to define immune equilibrium in mice.^3^ Stable equilibrium using *d*106S-IL12 was established in a large cohort of mice, which were rerandomized at day 40 into new groups to receive either anti-CD4, anti-CD8, anti-IFNγ, anti-NK1.1, or isotype control depleting antibodies every 3 days while continuing to receive *d*106S-IL12. Cell depletions were confirmed by flow cytometry (Figure S5). Interestingly, we found that IFNγ blockade was the only depletion that significantly altered tumor growth and overall survival (Figure 4C). Although there was some stochastic outgrowth of isotype-treated tumors, there was no difference in tumor sizes or survival of these mice compared with mice depleted of CD4^+^ T cells, CD8^+^ T cells, or NK cells (Figure 4C).

We confirmed this dependence on IFNγ for stable tumor control using IFNγ-deficient mice. These mice failed to adequately control B16^Nectin1^ tumors treated with *d*106S-IL12 (Figure 4D), suggesting that IFNγ was necessary not only for maintenance but also for establishment of equilibrium. TCRa^−/−^ mice, which lack CD4^+^ and CD8^+^ T cells, showed an intermediate phenotype in which tumor growth was still slowed significantly over PBS treatment (Figure 4D). This intermediate phenotype argued that αβ T cells play a role in stabilizing tumor growth and establishing equilibrium, but they are not uniquely required for antitumor immunity during *d*106S-IL12 treatment. Combining CD4, CD8, and NK1.1 depleting antibodies at day 40 led to rapid outgrowth of tumors and poor survival of mice (Figure 4E). This suggested that these cell types played complementary roles, likely through their shared ability to secrete IFNγ, which is consistent with our findings in RAG2^−/−^ mice, which lack not only αβ but also γδ T cells, another potential source of IFNγ.^60^

Consistent with the importance of IFNγ in our model of equilibrium, transcriptomic data from The Cancer Genome Atlas (TCGA) were analyzed, and a positive association was found between IL-12, IFNγ-stimulated gene expression, and increased survival in melanoma patients (Figures S6A and S6B). Previous analysis has shown that cytolytic activity score (CYT; expression of *Gzma* and *Prf1*) is associated with increased survival benefit in cancer patients.^61^ We observed that, indeed, melanoma patients with higher CYT fared better, as did patients with higher expression of IFNγ-response genes (Figure S6C).

### Direct T cell cytotoxicity is not required to control tumors treated with IL-12 virotherapy

**Figure 5.**
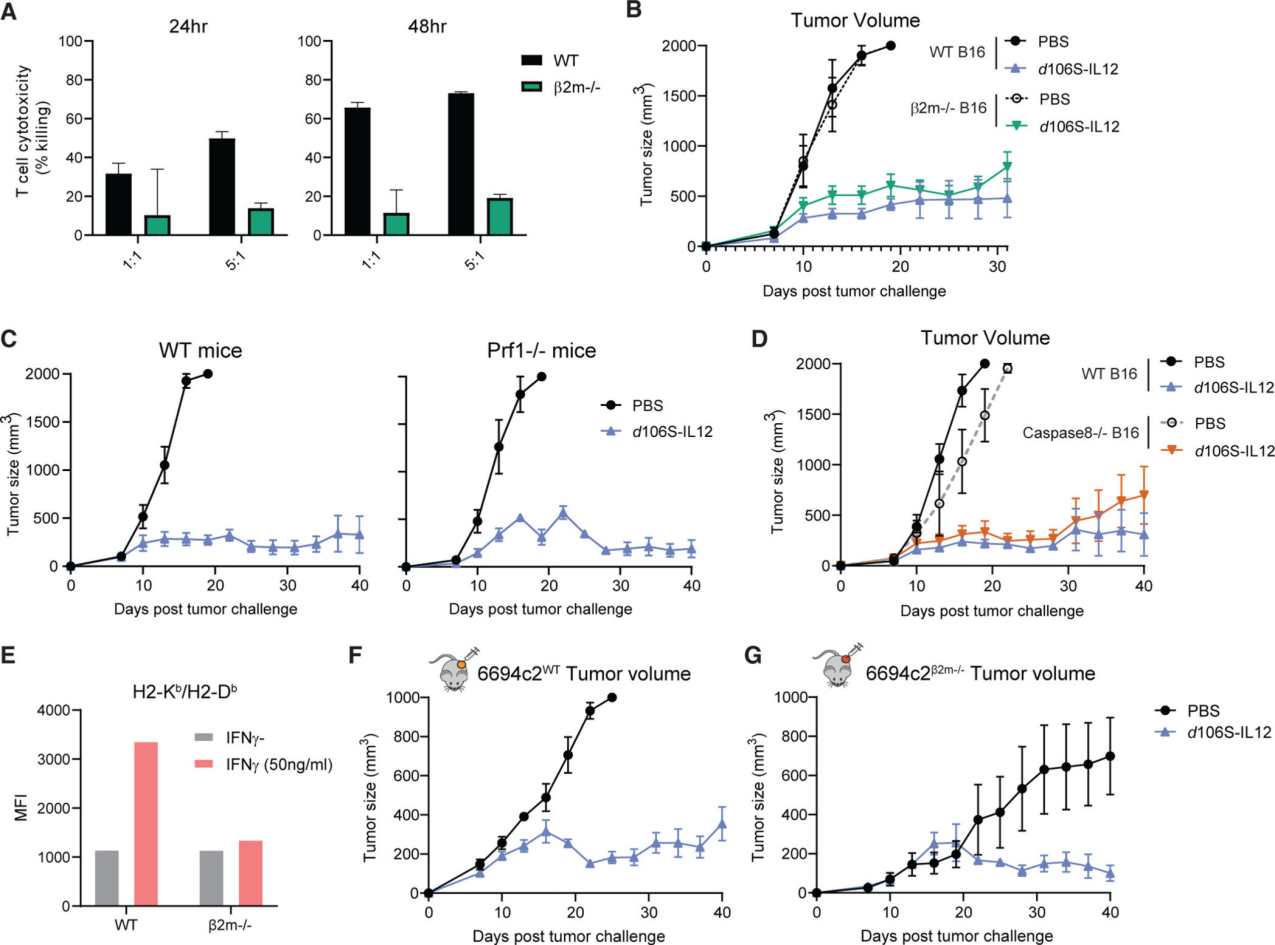
*d*106S-IL12 therapy controls tumors independent of direct T cell cytotoxicity (A) TRP1^high^ effector CD8^+^ cells were co-cultured for 24–48 h with IFNγ-stimulated, CFSE-labeled B16^Nectin1^ wild-type or *β2m* CRISPR-Cas9 knockout cells, and cytotoxicity was measured by fluorescent confluence using a Celigo image cytometer. See also Figure S7. (B) Mice bearing wild-type or *β2m*^−/−^ B16^Nectin1^ tumors were injected every 3 days starting at day 7. (C) Wild-type or perforin (*Prf1*)-deficient mice were challenged with B16^Nectin1^ tumors and treated every 3 days (N = 5 per group for WT; N = 4 per group for *Prf1*^−/−^). (D) Mice bearing wild-type or Caspase8^−/−^ B16^Nectin1^ tumors were treated every 3 days starting at day 7. (E) 6694c2^WT^ and 6694c2^β2m—/—^ murine pancreatic cancer cells were cultured for 24 h with or without IFNγ (50 ng/mL), and H2-K^b^/D^b^ (MHC-I) expression was measured by flow cytometry. MFI, mean fluorescence intensity. (F and G) 6694c2^WT^ cells (F) or 6694c2^β2m—/—^ cells (G) were implanted subcutaneously, and treatment began on day 7 and proceeded every 3 days. N = 5 for PBS, 5/10 for *d*106S-IL12 groups (6694c2/B16, respectively) unless otherwise specified. Values are mean ± SEM. Survival groups were compared using a log-rank test.

Although we had seen that CD8^+^ T cells were dispensable after equilibrium establishment, we wished to test the role of cell-mediated cytotoxicity in generating tumor control induced by *d*106S-IL12. We therefore generated *B2m* CRISPR-Cas9 knockout B16 cells deficient for MHC class I expression, with *in vitro* growth and HSV infectivity similar to those of wild-type B16^Nectin1^ cells (Figures S7A and S7D). The β2m^−/−^ cells did not have detectable MHC class I on their cell surface (Figure S7G) and were resistant to antigen-specific CD8^+^ T cell killing *in vitro* (Figure 5A). When we implanted B16^Nectin1^ wild-type or β2m^−/−^ cells into mice, we observed that *d*106S-IL12 treatment controlled tumors regardless of MHC class I status (Figure 5B), indicating that direct antigen presentation to CD8^+^ T cells was not required for establishment of equilibrium.

Because we had seen that depletion of multiple cell types led to more tumor outgrowth, we tested whether loss of effector function by multiple cell types elicited a change in tumor control. We challenged wild-type or perforin-deficient (Prf1^−/−^) mice with wild-type B16^Nectin1^ tumors and observed equivalent levels of control elicited by IL-12 virotherapy (Figure 5C). Therefore, perforin/granzyme cytotoxicity from CD8^+^ or CD4^+^ T cells or NK cells was not required. Cytotoxic effector cells can also kill target cells through tumor necrosis factor α(TNFα) or other TNF-family death ligands, such as FasL. We therefore generated B16 *Casp8* knockout cells, which lack caspase 8 (Figures S7B, S7E, and S7H), critical for extrinsic apoptosis, and we observed that caspase-8-deficient tumor cells were still able to be controlled by *d*106S-IL12 therapy to the same degree as wild-type cells (Figure 5D), suggesting that this cytotoxic pathway was not required. Using previously established β2m^−/−^ 6694c2 pancreatic cancer cells^62^ (Figure 5E), we confirmed that even in the absence of direct CD8^+^ T cell recognition, *d*106S-IL12 could elicit stable tumor control of highly refractive pancreatic cancer (Figures 5F and 5G).

### IFNγ-insensitive tumors are resistant to checkpoint blockade, but not *d*106S-IL12

**Figure 6.**
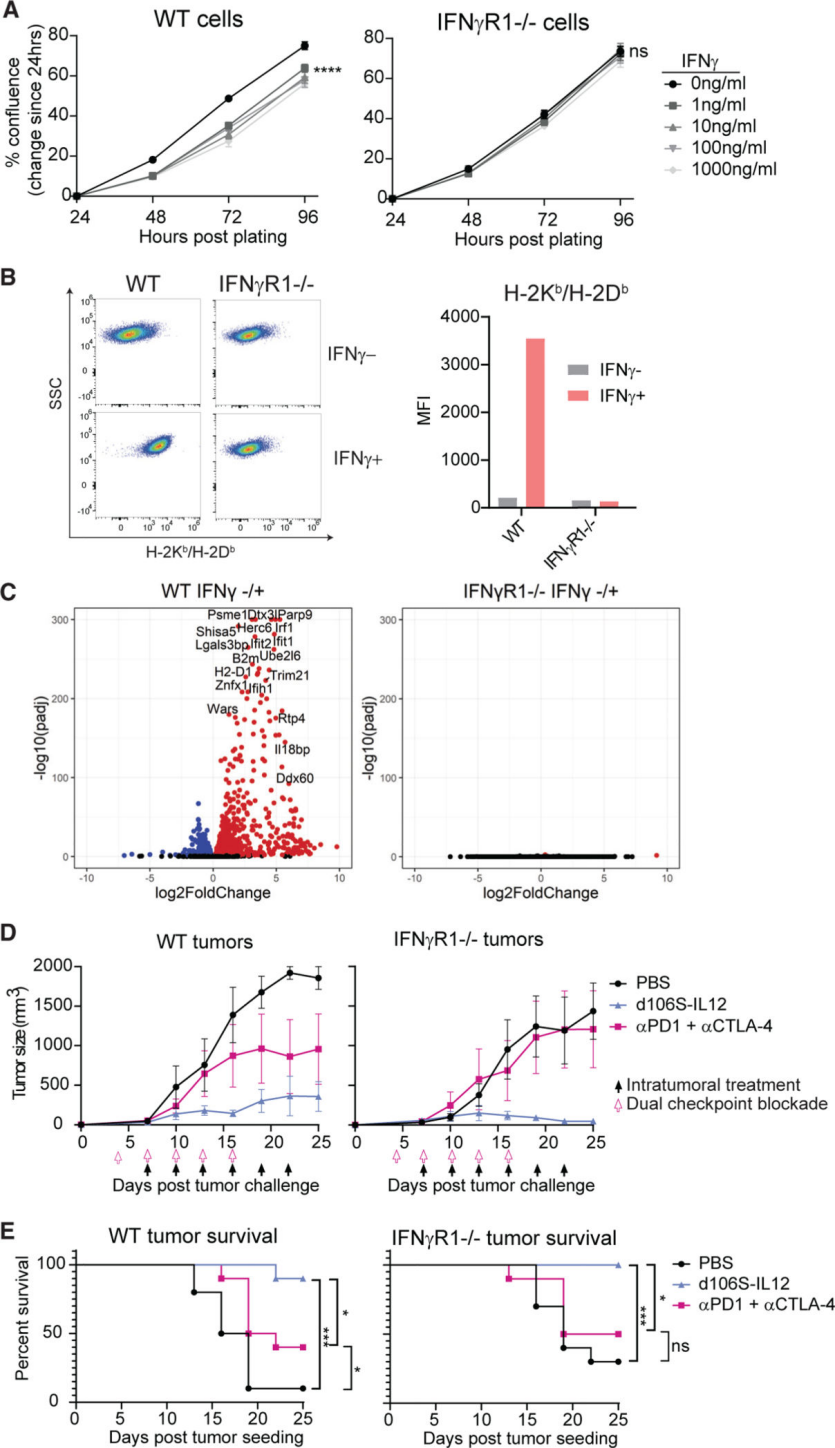
Tumors lacking IFNγ response are still controlled by *d*106S-IL12 therapy but not checkpoint blockade CRISPR-Cas9 gene editing was used to generate IFNγR1 (and STAT1; see also Figure S7) knockout B16^Nectin1^ cell lines. (A) Wild-type or IFNγR1^−/−^ cells were cultured with varying concentrations of IFNγ, and confluence was determined every 24 h by Celigo image cytometer. (B) Wild-type or IFNγR1^−/−^ cells were cultured *in vitro* with 50 ng/mL IFNγ and H2-K^b^/D^b^ (MHC-I) expression determined by flow cytometry 24 h later. Representative flow plots from multiple independent experiments. (C) RNA sequencing was performed on wild-type or IFNγR1^−/−^ cells cultured *in vitro* in the absence or presence (100 ng/mL) of IFNγ for 24 h (n = 3 per group). Differential gene expression was performed comparing IFNγ^−^ versus IFNγ^+^ samples within the same genotype. (D) Wild-type mice were challenged with either wildtype or IFNγR1^−/−^ B16^Nectin1^ tumors and treated with PBS or *d*106S-IL12 starting at day 7 or anti-PD-1^+^ anti-CTLA-4 antibodies (150 mg each) i.p. starting at day 4 and proceeding every 3 days (N = 5 mice per group). (E) Pooled survival across two experiments as in (D) (N = 10 mice per group). Values are mean ± SEM, N = 3 unless otherwise noted. Confluence was compared by two-way ANOVA with Dunnett’s multiple comparisons test. Survival groups were compared using a log-rank test. *p < 0.05; ***p < 0.001; ****p < 0.0001.

Based on the importance of IFNγ in establishing and maintaining stable tumor control in response to *d*106S-IL12 therapy, we hypothesized that large amounts of IFNγ could act directly on the tumor and slow its growth. To this end, we generated *IFNγr1* and *Stat1* CRISPR-Cas9 knockout B16^Nectin1^ cells that would be deficient in binding or signaling downstream of IFNγ, respectively. These cells had similar *in vitro* growth kinetics and HSV infectivity (Figures S7C and S7F). While wild-type cells exhibited slowed growth kinetics *in vitro* in the presence of IFNγ, IFNγR1^−/−^ (Figure 6A) and STAT1^−/−^ cells did not (Figure S7I). The knockout cells also did not upregulate MHC class I in response to IFNγ (Figures 6B and S7J). RNA sequencing of wild-type cells showed a robust upregulation of hundreds of genes in response to IFNγ stimulation *in vitro*, while the IFNγ-insensitive cells were transcriptionally unaffected (Figures 6C and S7K).

After validating that IFNγR1^−/−^ B16^Nectin1^ cells were truly insensitive to IFNγ-mediated effects, we challenged wild-type mice with either control or IFNγR1^−/−^ tumor cells, established palpable tumors, and began treatment with dual checkpoint blockade or IL-12 virotherapy. Checkpoint blockade typically fails to protect mice with tumors that are insensitive to IFNγ^6,11^ and, indeed, dual checkpoint blockade using anti-PD-1 and anti-CTLA-4 antibodies failed to protect mice with IFNγ-insensitive tumors while significantly protecting mice with wild-type B16^Nectin1^ tumors (Figures 6D and 6E). Surprisingly, all tumors treated with *d*106S-IL12 were controlled regardless of IFNγ-sensing ability (Figures 6D, 6E, and S7L). This suggested that despite IFNγ being critical for tumor control, this cytokine did not need to act directly on tumor cells to exert an effect.

### IFNγ can act directly on tumors or multiple host cell types to generate stable equilibrium

**Figure 7.**
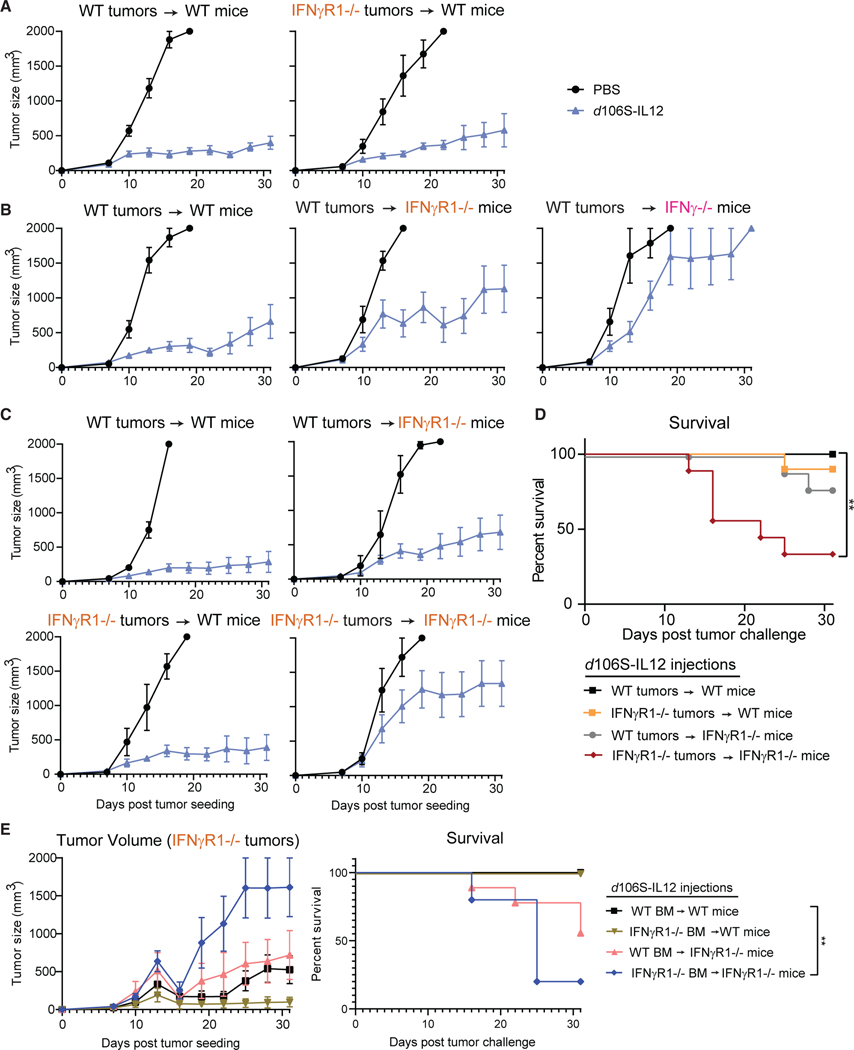
IFNγ can control tumors through actions on host or tumor cells (A) Wild-type mice bearing wild-type or IFNγR1^−/−^ B16^Nectin1^ tumors were injected every 3 days starting at day 7 (N = 5 for PBS, N = 10 for *d*106S-IL12 groups). (B) Wild-type B16^Nectin1^ tumors were inoculated into wild-type, IFNγR1^−/−^, or IFNγ^−/−^ mice and treated every 3 days (N = 5/10, 5/7, and 4/4 PBS/*d*106SIL12 groups, respectively). (C) Wild-type or IFNγR1^−/−^ mice were challenged with wild-type or IFNγR1^−/−^ tumors and treated every 3 days (N = 5/10 PBS/*d*106SIL12 for all groups except 5/9 for IFNγR1^−/−^ tumors into IFNγR1^−/−^ mice). (D) Survival from (C). Wild-type or IFNγR1^−/−^ bone marrow was used to inoculate irradiated wild-type or IFNγR1^−/−^ recipient mice. Mice were rested for several months before B16 IFNγR1^−/−^ tumor challenge and subsequent *d*106S-IL12 treatment (every 3 days beginning day 7). (E) Tumor volume and survival of bone marrow chimera mice challenged with B16 IFNγR1^−/−^ tumors. Groups from top to bottom: N = 10, 9, 9, and 5. Values are mean ± SEM. Survival groups were compared using a Bonferroni-corrected log-rank test. **p < 0.01.

As signaling of IFNγ in the tumor cells was found to be dispensable for equilibrium, we next sought to determine which host cells were responding. As expected based on our previous results, we confirmed that IFNγR1-deficient tumors were still maintained in equilibrium when treated with IL-12 virotherapy (Figure 7A). We hypothesized that IFNγ was acting on host cells to control tumors. Surprisingly, when we challenged IFNγR1^−/−^ mice with wild-type tumors, we discovered that these mice could still largely control their tumors, suggesting that tumor sensing of IFNγ was now controlling tumor growth (Figure 7B).

Based on these findings, we concluded that IFNγ could act on the tumor cells directly and/or on host cells to indirectly control tumors via downstream effects of IFNγ. Either pathway could be compensatory when the flow of IFNγ to tumor or host cells was disrupted by IFNγ-sensing defects. To test this multimodal hypothesis, we challenged wild-type or IFNγR1^−/−^ mice with wild-type or IFNγR1^−/−^ tumors. Indeed, wild-type mice bearing wild-type tumors controlled their tumors to a similar extent as wild-type mice bearing IFNγR1^−/−^ tumors or IFNγR1^−/−^ mice bearing wild-type tumors (Figures 7C and 7D). However, when neither tumors nor the host could sense IFNγ, the stable control of tumors was lost and tumors grew progressively (Figures 7C and 7D).

To understand which host cell types were involved in sensing of IFNγ to control IFNγR1^−/−^ tumors, we generated bone marrow chimeric mice of irradiated wild-type or IFNγR1^−/−^ background bearing wild-type or IFNγR1^−/−^ bone marrow. We challenged these mice with IFNγR1^−/−^ tumors to ensure that IFNγ could only act on the host cells. Interestingly, IFNγR1^−/−^ tumors were able to be controlled in all groups except IFNγR1^−/−^ mice with IFNγR1^−/−^ bone marrow (Figure 7E), which phenocopy the total IFNγR1-deficient mice that did not control tumors well (Figures 7C and 7D). This indicates that both hematopoietic and non-hematopoietic cells can respond to IFNγ to control tumors, suggesting a highly compensatory, multimodal mechanism of tumor control during equilibrium in which IFNγ is a key node.

## DISCUSSION

In this study, we have generated a highly reproducible model of therapy-induced tumor equilibrium. We discovered that IFNγ is the central player in both establishment and maintenance of equilibrium through a variety of redundant mechanisms. Most current immunotherapies rely on direct CD8^+^ T cell killing, with the consequence that loss of antigen presentation or defects in the IFNγ-sensing pathway allows tumors to escape CD8^+^ T cell-mediated immune pressure. Here we found that IL-12 virotherapy was effective across multiple settings in which the tumor cells were not directly recognizable by CD8^+^ T cells, including loss of β2m. The perforin-granzyme pathway and caspase-8-dependent death receptor pathways were likewise dispensable. This suggests that although CD8^+^ T cell direct cytotoxicity may play a role in initial tumor control, immune equilibrium is not maintained by CD8^+^ T cells directly recognizing tumor cells.

Numerous studies have demonstrated the importance of IFNγ for immunosurveillance and control of tumors,^3,60,63^ although it has been less clear how IFNγ functions in equilibrium. Here we confirm the importance of IFNγ for controlling tumors but demonstrate that its protective effects are not mediated solely through its direct action on tumors to slow their growth and increase antigen presentation. IFNγ can also serve to coordinate an immune response against tumors. IFNγ can enhance the activation of macrophages to express nitric oxide synthase to release reactive nitrogen oxide species and increase their phagocytic function. Nitric oxide species may damage tumor cells^64^ but also can promote immunosuppression that may dampen tumor control.^65,66^ Tumor cell control by macrophages can also be due to enhanced phagocytosis. Work from our lab has shown that in the context of cIAP1/2 antagonism, T cells can repolarize macrophages to phagocytose live tumor cells in a manner independent of direct T cell recognition of tumor MHC or IFNγ sensing by the tumor.^62^ Through scRNA-seq of the tumor microenvironment, we saw a strong IFNγ response generated by many cells, but most notably by macrophages.

While macrophages may play some role in controlling tumors in our system, the bone marrow chimera experiment suggests that both hematopoietic and non-hematopoietic cell types can sense and respond to IFNγ to control IFNγ-insensitive tumors. The antiangiogenic properties of IFNγ are well known and may be a possible mechanism for non-hematopoietic cells to control tumors in response to the cytokine.^13,14,67^ Additionally, tissue-resident macrophages arise from self-renewing populations independent of bone marrow hematopoiesis and are radioresistant.^68^ Thus, wild-type mice with IFNγR1^−/−^ bone marrow could still have tissue-resident macrophages capable of responding to IFNγ to control IFNγ-insensitive tumors when treated with IL-12 virotherapy. Wild-type mice with IFNγR1^−/−^ bone marrow would also still have stromal vasculature that could respond to IFNγ and reduce blood flow, causing tumors to be better controlled.^14^ Although we did not examine the vasculature in these chimeric mice, wild-type mice did not have a reduction in CD31^+^ vascular endothelial cells with *d*106S-IL12 treatment.

IFN-response genes are induced by TNFα, IFNβ, and IFNγ. A previous study found that PD-1 resistance driven by either IFNγ-sensing defects or β2m^−/−^ tumors could be overcome by co-administration of anti-PD-1 with either a TLR9 agonist or an IL-2 agonist, respectively.^11^ TLR9 agonism induces type I IFN production that signals through TNFΑR on tumor cells and has overlapping function with IFNγ.^11^ Although our virotherapy induces a type I IFN response, blockade of TNFΑR in mice only affected the initial set point size of tumors but does not affect establishment of immune equilibrium, despite repeated administration of virotherapy and continual type I IFN production. Outgrowth of tumors in IFNγ^−/−^ and RAG2^−/−^ mice, as well as IFNγR1^−/−^ mice with IFNγR1^−/−^ tumors, was delayed relative to control-treated tumors, which was likely due to type I IFN-mediated effects from the viral vector, although these tumors ultimately did not enter stable equilibrium. We therefore conclude that IFNγ has a central role in establishment and maintenance of equilibrium that is not recapitulated by IFNα or IFNβ.

The concept of equilibrium assumes an equal balance between tumor cell proliferation and tumor cell death. One means of achieving equilibrium is to induce tumor cell quiescence, thereby reducing the proliferation to zero. Tumor cell quiescence has been associated with resistance to CD8 T cell-mediated immunotherapy and can be induced by IFN signaling.^69^ Here we were surprised to find actively proliferating tumor cells in late-stage equilibrium tumors, suggesting that therapy-induced immune equilibrium is an active state of tumor control. Although the overall levels of proliferating cells were lower, partially due to large areas of necrotic tissue, we observed an equal ratio of proliferating cells to those undergoing apoptosis. The fact that tumors grow progressively upon cessation of virotherapy and the fact that tumor cell loss of IFN sensing is inadequate to prevent establishment of equilibrium support the hypothesis that immune equilibrium can occur by means other than IFNγ-induced tumor cell quiescence.

IFNγ may have evolved as a multimodal cytokine to protect the host against acute and chronic threats from transformed or infected cells. Although the antiviral effects of type I IFNs are best studied, IFNγ also has antiviral effects. In both acute and chronic hepatitis C virus infection, IFNγ levels are correlated with decreased viral load,^70^ and patients without IFNγ-expressing CD8^+^ T cells fare worse than those with IFNγ^+^ CD8^+^ T cells.^71^ Hepatitis B virus can be controlled directly by IFNγ acting on hepatocytes to reduce infection in a non-cytolytic manner.^72^ IFNγ was found to be the dominant IFN expressed in recurrent HSV-2 lesions, causing high levels of ISG expression by epidermal cells, which dampens infection.^73^ In babies born to human immunodeficiency virus (HIV-1)-positive mothers, IFNγ responses in the breast milk were associated with a 70% reduction in infant HIV acquisition.^74^ Additionally, IFNγ levels are negatively correlated with HIV-1 viral set point.^75^ Thus, IFNγ serves as a multipurpose cytokine: capable of initiating and integrating antiviral and antitumor responses through innate and adaptive immune cells as well as being directly antiviral and antitumor against affected cells.

Because the effects of downstream IFNγ are multimodal, involving several cell types that can possibly secrete the cytokine, including CD8^+^ and CD4^+^ T cells and NK cells as well as many cell types that can respond to IFNγ to control tumors, escape from equilibrium is more difficult to achieve. However, escape still does occur stochastically in some mice, modeling the clinical situation in humans who experience disease recurrence after long periods of tumor control. Acquired resistance has been linked with the inability of tumors to respond to IFNγ in patients,^9,10^ suggesting that host cell responsiveness to IFNγ may be impaired within some human tumors due to immune exclusion or other heterogeneities. Escape from equilibrium is a huge unmet clinical problem, and our model provides an opportunity to study these and other questions related to the mechanisms behind therapy-induced equilibrium, with the ultimate goal of understanding how to overcome equilibrium and effect tumor elimination. The seminal study by Koebel et al. demonstrated in mice that immune equilibrium was a distinct phase from elimination and escape, showing that low-dose carcinogen exposure can produce tumors held in check by the immune system for long periods of time.^3^ Only subsequent depletion of both CD4^+^ and CD8^+^ T cells, IFNγ, IL-12, or a combination of cellular and cytokine blockade resulted in escape and the outgrowth of these stable tumors. Because combination blockades were shown to be no more effective at disrupting equilibrium than individual depletions, it can be inferred that these factors control tumors in redundant ways, likely through IFNγ-mediated effects.^3^ Here, we demonstrate the central role of IFNγ in the establishment and maintenance of equilibrium in the context of immunotherapy. We demonstrate that in the absence of multiple pathways including type I IFN, tumor sensing of IFNγ, CD8^+^ T cell direct recognition of tumor MHC class I, perforin-granzyme-mediated cytotoxicity, and extrinsic death receptor signaling such as Fas/FasL IFNγ can still ultimately orchestrate long-term durable tumor control.

## STAR★METHODS

### RESOURCE AVAILABILITY

#### Lead contact

Further information and requests for resources and reagents should be directed to and will be fulfilled by the lead contact, Stephanie Dougan (stephanie_dougan@dfci.harvard.edu)

#### Materials availability

All unique materials in this study may be made available upon request from the lead contact.

#### Data and code availability

- Bulk and single-cell RNA-sequencing data are available at the Gene Expression Omnibus (GEO) under accession numbers: GSE212829 and GSE222795. Links can be found in the key resources table.
- This paper does not report original code.
- Any additional information required to reanalyze the data reported in this manuscript is available from the lead contact upon request.

### EXPERIMENTAL MODEL AND SUBJECT DETAILS

#### Cell culture and Nectin-1 generation

B16F10 and HEK293T cells were purchased from American Type Culture Collection. B16 cells were transduced with a previously described lentivirus encoding human nectin-1 (hNectin-1 vector a gift from Dr. Antonio Chiocca.^45^ Following hygromycin selection (500mg/mL), expression of nectin-1 was validated by infecting B16^Nectin1^ cells with *d*106S virus and selecting clones that were GFP-positive by flow cytometry analysis (FACSCalibur). KPC 6694c2 cells were derived from an LSL-Kras^G12D^;p53+/floxed, Pdx-cre, YFP-floxed mouse as described previously and were a gift from Dr. Benjamin Stanger.^54^ The Vero-based E11 complementing cell line,^43,76^ which expresses ICP27 and ICP4, was used to grow *d*106S virus. K28, K29, A375, UACC-257, and SK-MEL-2 patient melanoma cell lines were a kind gift from Dr. Frank Hodi (Dana-Farber Cancer Institute). B16, E11, and patient melanoma cells were cultured in DMEM with 10% heat-inactivated FBS and 1% PenStrep. Murine bone-marrow derived dendritic cells (BMDCs) were obtained as described previously.^57^ Briefly, bone marrow aspirates were cultured with complete RPMI media (with 10% heat-inactivated FBS, 2mM glutamine, 1% PenStrep, 1mM sodium pyruvate, 0.1mM β-ME) supplemented with 20 ng/mL each recombinant mGM-CSF and mIL-4 (Peprotech). Fresh media was added every 2 days for 6 days. BMDCs were used for co-culture experiments at day 6–8 of differentiation.

#### Animals

C57BL/6J mice aged 6 to 8 weeks, RAG2^−/−^ (#008449), TCRα^−/−^ (#002116), IFNγ^−/−^ (#002287), and IFNγR1^−/−^ (#003288) mice were purchased from Jackson Laboratories. TRP1^high^ mice (deposited as jax #030958) were previously generated.^57^ All mice were housed in the Dana-Farber Cancer Institute Animal Resources Facility. Roughly 80% of mice used were females. All animal experiments were performed in accordance with the DFCI IACUC-approved protocols (#14–019 and #14–037) and are in compliance with the NIH/NCI ethical guidelines for tumor-bearing animals.

### METHOD DETAILS

#### Plasmids

The coding sequence for the murine IL-12 fusion protein was cloned from Tandem p40p35, a gift from Nevil Singh; Addgene plasmid #108665. The sequence was amplified with XhoI_IL12_Fow and NotI_IL12_Rev primers (IDT). The PCR product was cloned into the pd27B shuttle plasmid by XhoI/NotI (NEB) digestion, followed by Quick Ligation (NEB). The correct insertion into the shuttle plasmid was confirmed by Sanger sequencing (Harvard Biopolymers Facility).

#### CRISPR-Cas9 knockouts

STAT1, IFNγR1, and B2m mutants were generated using pSpCas9(BB)-2A-Puro (PX459) V2.0 (Addgene #62988).^78^ Single-guide RNAs (sgRNA) were cloned into this vector using BbsI (NEB) and T4 ligation (NEB). Cells were transfected with sgRNA-containing plasmids using Lipofectamine Stem reagent (ThermoFisher), selected with puromycin (2 μg/mL), FACS-sorted for purity, and absence of protein was confirmed by flow cytometry following addition of murine IFNγ (50 ng/mL) (Peprotech) to the media. Caspase8 mutants were generated using the lentiCRISPRv2 plasmid (Addgene # 52961) and sgRNAs cloned in using BsmBI (NEB) digestion, followed by Quick Ligation (NEB). Lentivirus was generated by transfection of HEK293T cells with lentiCRISPRv2 (Addgene #62961),^79^ pVSVG (Addgene #8454),^80^ and psPAX2 (Addgene #12260) using Lipofectamine LTX reagent (ThermoFisher). Supernatants were collected 48 h after transfection, filtered through a 0.45μM pore, and used to infect tumor cells; puromycin was used to select cells. Knockout was confirmed by Western blot as described previously^51^ using anti-caspase 8 primary antibody (Cell Signaling #4927) and anti-GAPDH (abcam 8245).

#### Recombinant herpes simplex virus generation

The pd27-IL12 shuttle plasmid was linearized by SwaI (NEB) digestion. E11 complementing cells that express Cas9 and sgRNAs targeting the native HSV genome^76^ were co-transfected with linear pd27B-IL12 and infectious *d*106S DNA. Infectious *d*106S DNA was isolated as described previously.^42^ The progeny viruses were harvested and fluorescence-negative plaques were isolated and purified three times. Each plaque isolate was analyzed for evidence of IL12 insertion and subsequent lack of GFP by PCR. Viral stocks of both *d*106S and *d*106S-IL12 were grown and titered on E11 complementing cells.^43^ B16^Nectin1^ cells were infected at varying multiplicities of infection (MOIs) with *d*106S or *d*106S-IL12. Twenty-four hours following infection, the supernatant was harvested, and IL-12 production measured by IL12p40 ELISA (BioLegend).

#### BMDC co-culture and mRNA expression analysis

For mRNA expression analysis of co-culture experiments, B16^Nectin1^ cells were first infected at MOI 10 with *d*106S or *d*106S-IL12. Infected B16 cells were washed multiple times with media before addition of BMDCs for co-culture. Additionally, both B16^Nectin1^ and BMDCs in isolation were infected with *d*106S or *d*106S-IL12 at MOI 10 without co-culture. Total RNA was extracted from single and co-culture infections using the Qiagen RNeasy Kit and DNase treated with the DNA-free kit (Ambion). DNase-treated RNA was reverse-transcribed using the High Capacity cDNA Reverse Transcription kit (ThermoFisher). cDNA was quantified using the Fast SYBR Green Master Mix and StepOne Real-Time PCR System (ThermoFisher). qPCR reactions were carried out in triplicate. Samples were normalized to 18S rRNA.

#### DNA extraction for virus quantification

Mice bearing B16^Nectin1^ tumors were bled retroorbitally into a tube containing 0.5M EDTA to prevent clotting. *d*106S-IL12 virus was spiked into blood from untreated mice bearing B16^Nectin1^ tumors as a positive control. DNA was extracted from the blood using the Qiagen Blood/Tissue DNA extraction kit. Two RAG2^−/−^ mice near endpoint were sacrificed the day following a final injection of *d*106S-IL12. Their tumors were placed into DMEM containing 1% FBS, homogenized, freeze-thawed three times, filtered, and centrifuged at 14,000 x g for 10 min to yield a clarified viral lysate. DNA was extracted from this lysate as described above. qPCR reactions were carried out in triplicate to quantify copies of viral ICP8 DNA. Samples were normalized to GAPDH DNA.

#### Chemokine/cytokine analysis

B16^Nectin1^ tumors collected on day 10 and day 17 following one or four intratumoral injections, respectively, were snap frozen in liquid nitrogen. Frozen tumors were ground into a powder and tissue homogenate generated by addition of ice-cold RIPA lysis buffer (150mM NaCl, 1% NP-40, 0.5% sodium deoxycholate, 0.1% SDS, 25mM Tris) and 1:100 protease/phosphatase inhibitor cocktail (ThermoFisher). Homogenate was incubated shaking for 30 min at 4^◦^C and subsequently centrifuged at 14,000 x g for 15 min to yield clarified lysate. The clarified supernatant was subjected to a 32-plex cytokine/chemokine array (Eve Technologies). The protein concentrate of lysate was determined using the Micro BCA Protein Assay Kit (ThermoFisher) to normalize samples to contain an equal amount of protein.

#### Tumor inoculations and *in vivo* experiments

B16^Nectin1^ and 6694c2 cells were screened prior to *in vivo* use for murine pathogens, including mycobacteria (Charles River Laboratories). B16^Nectin1^ or 6694c2 cells were cultured until 80–90% confluent, trypsinized, washed and resuspended in Hank’s balanced salt solution (HBSS) at 2 × 10^6^ cells/mL or 8 × 10^5^ cells/mL, respectively. Mice were shaved and 250μL (5 × 10^5^,2 3 10^5^) cells were injected subcutaneously in the left flank, or right flank for bilateral/rechallenge experiments. Before injections, mice were randomized into treatment groups. Weight change was measured relative to the day before treatment began. For survival experiments, tumor size was measured every three to four days by precision calipers. Mice were euthanized when tumor volume exceeded 2,000 mm^3^ or tumors developed ulcerations. Intratumoral injections of 30μL PBS, *d*106S (1.5 × 10^7^ PFU/30μL), or *d*106S-IL12 (1.5 × 10^7^ PFU/30μL) were performed every three days.

#### Histopathology, immunohistochemistry, and immunofluorescence

B16^Nectin1^ tumors and surrounding tissue were excised and submerged in 10% formalin. Tissue was paraffin embedded, sectioned, and stained with hematoxylin and eosin by the Harvard Rodent Histopathology Core. For immunohistochemistry, unstained tissue sections were stained with CD31, cleaved caspase 3, or Ki67, and counterstained for nuclei (hematoxylin) by the Harvard Specialized Histopathology Core. For immunofluorescence, unstained tissue sections were dewaxed and rehydrated; antigen retrieval was performed in 10mM sodium citrate. Following blocking, sections were stained with anti-Ki67 (Abcam 15,580). Secondary staining was performed with goat anti-rabbit AF488 (ThermoFisher A-11008) and samples counterstained with DAPI. All sections were imaged with an Olympus 1X73 inverted fluorescence microscope and Olympus DP74 color camera using Olympus cellSens Imaging software. Stitched images were processed with the Olympus cellSens Imaging software. Color deconvolution of the red chromogen staining was performed with ImageJ/Fiji.^81^ CD31, Ki67, or cleaved caspase 3 area was determined by ImageJ/Fiji analysis of stitched sections for percent area of red chromogen within the tumor area (including necrotic tissue).

#### Single-cell RNA-sequencing processing

Tumors were collected and processed as described previously.^90^ Briefly, the tumor was excised, manually dissociated with scissors, and incubated in RPMI with tumor digestion enzymes (Miltenyi tumor dissociation kit, Cat. No. 130–096-730) for 30 min at 37^◦^C. Digested tumors were filtered through a 40μM strainer and cells were collected and resuspended in FACS buffer (PBS with 2% FBS). Cells were counted with a hemacytometer and equal portions of cells from each mouse in a sample group were pooled before staining with anti-CD45 (Biolegend clone 30-F11) and ZombieNIR (Biolegend #423105) and sorted for ZombieNIR^−^ CD45^+^ events into collection buffer (RPMI 1640, 25mM HEPES, 10% FBS) on a BD FACS Aria II at the Dana Farber Flow Sorting Core. Sorted cells were washed one time with 5mL PBS +0.05% ultrapure BSA, counted via hemacytometer, and resuspended at a concentration of 1000 cell/μL. Cell suspensions were loaded onto a 10x Chromium instrument with the Single Cell A Chip per manufacturer’s protocol with a targeted recovery of 5000 cells/sample. Library preparation was performed with the Chromium Single Cell 5′ Library and Gel Bead Kit (#1000006), and samples were sequenced on an Illumina HighSeq instrument with 2 × 150bp sequencing.

#### Single-cell RNA-sequencing analysis

The 10x Cellranger pipeline (v6.1.0) was used to align reads to the Mm10 reference genome and generate a single-cell feature count matrix for each library using default parameters. The count matrices were imported for downstream analysis into R using the *Seurat* (v4.0.2) package. The *DropletUtils* (v1.10.3) package^82^ was used to exclude empty droplets with an FDR <0.01. Genes expressed in fewer than 3 cells were discarded from further analysis. Barcodes were classified as cells if they satisfied the following criteria: minimum of 500 genes per cell and percentage of mitochondrial reads less than 3 MADs from the median. Counts from all samples were merged into one matrix, split by sample, then log-normalized individually. 2000 variable features were identified for each sample, from which 2000 integration anchors were identified and used to integrate the datasets together to minimize batch effects.^83^ The integrated dataset was then scaled and subjected to dimensionality reduction using Principal Component Analysis (PCA) of the integration anchors. Uniform Manifold Approximation and Projection (UMAP) embedding was generated from the top 14 dimensions of the PCA. Clusters were identified first by constructing a Shared Nearest Neighbor (SNN) graph based on each cell’s 20-nearest neighbors and then applying modularity refinement with the Louvain algorithm. Markers for each cluster were identified by comparing expressions using the ‘FindAllMarkers’ function.

The average foldchange between gene expression in the PBS vs. *d*106S-IL12 samples was calculated with the ‘FoldChange’ function and used for Gene Set Enrichment Analysis (GSEA). GSEA was performed with the *fgsea* package (v.1.16.0), using the Molecular Signature (v7.2) Hallmark gene signatures.^84^ Genes from the “Hallmark Interferon Gamma Response” were scaled and summed for each cell to visualize expression across samples. T cells were subclustered based on expression of at least two CD3 chains (*Cd3d*, *Cd3e*, *CD3g*), but no expression of *Csf1r*, *Cd79a*, *Lgmn*, or *Lyz2*. The top 2000 variable genes from these T cells were used for PCA and the top 12 PCA dimensions were used for UMAP generation. The R packages *Seurat* or *ggplot2* (v3.3.6) were used for visualization of scRNA-seq data.

#### Antibody blockade in vivo

For type I IFN blockade mice were injected i.p. with 200mL (100mg) of anti-TNFΑR1 (BioXCell Clone MAR1–5A3) starting on day seven and continuing every three days until day 34. For equilibrium disruption experiments: at day 40 surviving mice treated with *d*106S-IL12 were rerandomized and injected with 200μL (100μg) of either: anti-CD4 (BioXCell Clone GK1.5), anti-CD8α (BioXCell Clone 2.43), anti-IFNγ (BioXCell Clone H22), anti-NK1.1 (BioXCell Clone PK136), isotype antibody control (BioXCell Clone LTF-2) or anti-CD4 + anti-CD8α + anti-NK1.1 (100μg each) starting on day 40 every three days for a total of seven injections while continuing to receive *d*106S-IL12 treatment. For checkpoint blockade experiments: at day four post tumor challenge mice received 150μL (150μg) each of anti-PD-1 (BioXCell Clone RMP1–14) and anti-CTLA-4 (BioXCell Clone 9D9) every three days for five total injections.

#### Bone marrow chimeras

Wild-type or IFNγR1^−/−^ recipient mice were irradiated with 4.5 Gy using a ^137^Cs source, rested for 5 h and irradiated with another 4.5 Gy. Irradiated mice were placed on Baytril-water for 14 days. Wild-type or IFNγR1^−/−^ mice donor mice were sacrificed, and bone marrow collected. Bone marrow with matching genotypes was pooled and evenly distributed among irradiated recipients by tail vein injection 8 h following second irradiation. Bone marrow chimeras were housed for several months prior to tumor challenge to ensure survival following reconstitution.

#### *Ex vivo* cultures

Two mice bearing endpoint tumors not responding to *d*106S-IL12 therapy were sacrificed. Their tumors were excised, manually dissociated with sterile scissors in a biosafety cabinet, and filtered through a 40mM strainer into DMEM containing 10% heat-inactivated FBS and 1% PenStrep. Each tumor was diluted successive times to generate a non-confluent monolayer. Cells were washed and media changed every one-two days; confluent wells were trypsinized and replated until a homogeneous monolayer was apparent (about 1–2 weeks). Cells were used in downstream infection and flow cytometry experiments after >1 week of culture.

#### CD8 cytotoxicity

CD8 cytotoxicity assays were performed as previously described.^91^ Briefly, CD8 T cells from TRP1^high^ Mice^57^ were isolated with EasySep Mouse CD8+ T cell Isolation Kit (StemCell), resuspended in complete RPMI +100U/mL hIL2 (Peprotech #200–02) and stimulated with CD3/CD28 beads Dynabeads (Thermofisher #11456D) for 48 h. Target B16^Nectin1^ or *ex vivo* cultures were plated in complete DMEM with 50 ng/mL IFNγ (Peprotech #315–05) on the day before co-culture. On day seven following T cell isolation, CD8 T cells were plated at various E:T ratios with or without the presence of tumor cells. 24 h following co-incubation of T cells with tumor cells, media was carefully removed and cell viability was assessed using CellTiterGlo (Promega). Relative luminescence was normalized to wells containing the same number of T cells without tumor cells present. For T cell cytotoxicity comparing wildtype B16^Necti1^ to β2m^−/−^ B16^Nectin1^, tumor cells were stained with 5μM CFSE prior to plating with 50 ng/mL IFNγ; T cell cytotoxicity was measured as fluorescent confluence loss comparing T cell containing wells to wells with tumor cells only using a Celigo image cytometer (Nexcelom 200-BFFL-5c).

#### Cell growth

B16^Nectin1+^ knockout cell line growth was compared to B16^Nectin1+^ wildtype cells using a Celigo image cytometer. To compare IFNγ-mediated growth delay, wild-type, STAT1^−/−^, or IFNγR1^−/−^ B16^Nectin1^ cells were plated in 96-well plates with varying amounts of IFNγ present during plating. Confluence measurements were made every 24 h after plating in 96-well plates.

#### Flow cytometry

Tumors were processed for flow cytometry as above for scRNA-seq: manual dissociation, enzyme digest, filtering, followed by staining with flow cytometry antibodies as below. Peripheral blood or spleens were collected and ACK-lysed prior to staining with flow cytometry antibodies for 20 min at 4^◦^C. Cells were washed once with PBS and fixed with 1% formalin in PBS before analysis, which was performed on a Sony Biotechnology SP6800 Spectral Analyzer and analyzed with the Sony Biotechnology SP6800 Software and FlowJo (Tree Star, Ashland, OR). Flow cytometry antibodies used in this study were purchased from Biolegend:, anti-CD11b (clone M1/70), anti-CD11c (clone N418), anti-CD4 (clone GK1.5), anti-CD45 (clone 30-F11), anti-CD8 (clone 53–6.7), anti-Gr1 (clone RB6–8C5), anti-Ly6C (clone HK1.4), anti-Tbet (clone 4B10), anti-TIM3 (clone RMT3–23), anti-CD111(Nectin-1) (clone R1.302), anti-CD274 (PD-L1) (clone 10F.9G2), anti-IA/IE (clone M5/114.15.2), anti-Kb/Db (clone 28–8-6), anti-NK1.1 (clone PK136), and ZombieNIR (#423105). Intracellular staining for T-bet was performed with eBioscience Foxp3/Transcription Factor Staining Buffer Set (Invitrogen 00–5523-00).

#### Bulk RNA-sequencing

Wild-type, STAT1^−/−^, and IFNγR1^−/−^ B16^Nectin1^ cells were cultured *in vitro* for 24 h with 0 or 100 ng/mL IFNγ (Peprotech). Total RNA was extracted from cells of biological triplicates using the Qiagen RNeasy Plus Kit. Library construction and Illumina sequencing was performed by Genewiz. Quantification of transcripts from reads was performed using *Salmon* (v1.8.0)^85^ with the mouse GRCm39 reference genome and Gencode transcript release v27. The R package *tximport* (v1.18.0)^86^ was used to quantify transcript abundance and *DESeq2* (v1.30.1)^92^ was used to identify differentially expressed genes. Volcano plots were generated using *ggplot2* (v3.3.6).

#### TCGA analysis

TCGA transcriptomic and clinical data were obtained from cBioPortal website^88,89^ for melanoma patients. For each gene, patients were stratified into expression above or below the median and the hazard ratio and p value based on the log rank test was calculated using the *survival* (v3.2–13) and *survminer* (v0.4.9) R packages. *survminer* was also used to plot TCGA survival curves. To adjust for false discovery, p values were corrected with the Benjamini-Hochberg procedure. CYT status was calculated as above/below the median of the geometric mean of *GZMA* and *PRF1* genes.^61^ IFNγ-response was calculated as above/below the median of scaled and summed expression of genes in the Gene Ontology database terms GO0034341, GO0071346, and GO0060335 (response to type II interferon, cellular response to type II interferon and positive regulation of type II interferon-mediated signaling pathway, respectively).

### QUANTIFICATION AND STATISTICAL ANALYSIS

#### Statistical analysis

All data were analyzed with GraphPad Prism or R. All data were presented as mean with S.E.M. errors bars. Kaplan-Meier survival curves of mice were analyzed using the log rank test with a Bonferroni correction to adjust for multiple comparisons. Data were considered significant when p < 0.05; *p < 0.05, **p < 0.01, ***p < 0.001, ****p < 0.0001.

## Supplementary Material

1

2

## Figures and Tables

**Table:** KEY RESOURCES TABLE

REAGENT or RESOURCE	SOURCE	IDENTIFIER
Antibodies		

InVivoMAb anti-mouse IFNAR-1	BioXCell	#BE0241; RRID:AB_2687723
InVivoMAb anti-mouse CD4	BioXCell	#BE0003-1; RRID:AB_1107636
InVivoMAb anti-mouse CD8α	BioXCell	#BE0061; RRID:AB_1125541
InVivoMAb anti-mouse IFNγ	BioXCell	#BE0312; RRID:AB_2736992
InVivoMAb anti-mouse NK1.1	BioXCell	#BE0036; RRID:AB_1107737
InVivoMAb rat IgG2b isotype control, anti-keyhole limpet hemocyanin	BioXCell	#BE0090; RRID:AB_1107780
InVivoMAb anti-mouse PD-1 (CD279)	BioXCell	#BE0146; RRID:AB_10949053
InVivoMAb anti-mouse CTLA-4 (CD152)	BioXCell	#BE0164; RRID:AB_10949609
Pacific Blue^™^ anti-mouse/human CD11b Antibody	Biolegend	101,224; RRID:AB_755986
PE/Cyanine7 anti-mouse Ly-6G/Ly-6C (Gr-1) Antibody	Biolegend	108,416; RRID:AB_313381
Brilliant Violet 570^™^ anti-mouse Ly-6C Antibody	Biolegend	128,030; RRID:AB_2562617
Brilliant Violet 421^™^ anti-mouse CD4 Antibody	Biolegend	100,438; RRID:AB_11203718
Brilliant Violet 785^™^ anti-mouse CD8a Antibody	Biolegend	100,750; RRID:AB_2562610
APC anti-mouse CD11c Antibody	Biolegend	117,310; RRID:AB_313779
Brilliant Violet 421^™^ anti-T-bet Antibody	Biolegend	644,816; RRID:AB_10959653
APC anti-mouse CD366 (Tim-3) Antibody	Biolegend	119,706; RRID:AB_2561656
PE anti-human CD111 (Nectin-1) Antibody	Biolegend	340,404; RRID:AB_2174152
Brilliant Violet 711^™^ anti-mouse CD45 Antibody	Biolegend	103,147; RRID:AB_2564383
APC anti-mouse CD274 (B7-H1, PD-L1) Antibody	Biolegend	124,312; RRID:AB_10612741
Alexa Fluor ^®^488 anti-mouse I-A/I-E Antibody	Biolegend	107,615; RRID:AB_493524
PE anti-mouse H-2Kb/H-2Db Antibody	Biolegend	114,607; RRID:AB_313598
FITC anti-mouse NK-1.1 Antibody	Biolegend	108,705; RRID:AB_313392
Anti-GAPDH antibody [6C5]	Abcam	ab8245; RRID:AB_2107448
Caspase-8 Antibody (Mouse Specific)	Cell Signaling	#4927; RRID:AB_2068301
Anti-Ki67 antibody	Abcam	ab15580; RRID:AB_443209
Goat anti-Rabbit IgG (H + L) Cross-Adsorbed Secondary Antibody, Alexa Fluor^™^ 488	ThermoFisher	ThermoFisher A-11008; RRID:AB_143165

Bacterial and virus strains		

NEB ^®^ 5-alpha Competent E. coli (High Efficiency)	NEB	C2987H
HSV-1 *d*106S	Liu et al.^41^	N/A
HSV-1 *d*106S-IL12	This paper	N/A

Chemicals peptides and recombinant proteins		

UltraPure^™^ BSA (50 mg/mL)	ThermoFisher	AM2618
Lipofectamine LTX with Plus Reagent	ThermoFisher	15,338,100
Lipofectamine^™^ Stem Transfection Reagent	ThermoFisher	STEM00001
OptiMEM	ThermoFisher	31,985,062
HBSS, calcium, magnesium, no phenol red	ThermoFisher	14,025,126
HEPES (1 M)	ThermoFisher	15,630,080
Penicillin-Streptomycin (10,000 U/mL)	ThermoFisher	15,140,122
L-Glutamine (200 mM)	ThermoFisher	25,030,081
Recombinant Murine GM-CSF	Peprotech	315-03
Recombinant Murine IL-4	Peprotech	214-14
Recombinant Murine IFNγ	Peprotech	315-05
Recombinant Human IL-2	Peprotech	200-02
XhoI	NEB	R0146S
NotI	NEB	R0189S
BbsI	NEB	R0539S
SwaI	NEB	R0604S
BsmBI-v2	NEB	R0739S
T4 DNA ligase	NEB	M0202S
Puromycin Dihydrochloride	ThermoFisher	A1113803
Hygromycin B (50 mg/mL)	ThermoFisher	10,687,010
Ambion^™^ DNase I (RNase-free)	ThermoFisher	AM2222
NP-40 Surfact-Amps^™^ Detergent Solution	ThermoFisher	85,124
Sodium deoxycholate	ThermoFisher	89,904
Halt^™^ Protease and Phosphatase Inhibitor Cocktail (100X)	ThermoFisher	78,440

Critical commercial assays		

Zombie NIR^™^ Fixable Viability Kit	Biolegend	423,105
Dynabeads^™^ Mouse T-Activator CD3/CD28 for T cell Expansion and Activation	ThermoFisher	11456D
EasySep^™^ Mouse CD8+ T cell Isolation Kit	Stemcell Technologies	19,853
Quick Ligation^™^ Kit	NEB	M2200S
ELISA MAX^™^ Deluxe Set Mouse IL-12/IL-23 (p40)	Biolegend	431,604
RNeasy Mini Kit	Qiagen	74,106
High-Capacity cDNA Reverse Transcription Kit	ThermoFisher	4,368,814
SYBR Green Master Mix	ThermoFisher	4,309,155
Micro BCA^™^ Protein Assay Kit	ThermoFisher	23,235
Tumor Dissociation Kit, mouse	Miltenyi	130-096-730
10x Chromium Single Cell A Chip Kit	10x Genomics	PN-1000152
10x Chromium Single Cell 5^′^ Library and Gel Bead Kit	10x Genomics	PN-1000006

Deposited data		

Bulk RNA-sequencing	This paper	NCBI GEO: GSE212829
Single-cell RNA-sequencing	This paper	NCBI GEO: GSE222795

Experimental models: Cell lines		

B16F10 melanoma	ATCC	CRL-6475
B16F10-nectin1	This paper	N/A
B16F10-nectin1 Stat1-knockout	This paper	N/A
B16F10-nectin1 Ifngr1-knockout	This paper	N/A
B16F10-nectin1 B2m-knockout	This paper	N/A
B16F10-nectin1 Casp8-knockout	This paper	N/A
KPC c6694c2	Li et al.^54^	N/A
KPC c6694c2 B2m-knockout	Roehle et al.^62^	N/A
Vero E11	Samaniego et al.^43^	N/A
Vero E11 Cas9	Oh et al.^76^	N/A
K28; K29; UACC-257	Patient derived cell lines, Dana-Farber Cancer Institute	N/A
A375	ATCC	CRL-1619
SK-MEL-2	ATCC	HTB-68
HEK293T	ATCC	CRL-3216

Experimental models: Organisms/strains		

Mouse: C57BL/6J	Jackson Laboratory	RRID:IMSR_JAX:000,664
Mouse: B6.Cg-Rag2tm1.1Cgn/J	Jackson Laboratory	RRID:IMSR_JAX:008,449
Mouse: B6.129S2-Tcratm1Mom/J	Jackson Laboratory	RRID:IMSR_JAX:002,116
Mouse: B6.129S7-Ifngtm1Ts/J	Jackson Laboratory	RRID:IMSR_JAX:002,287
Mouse: B6.129S7-Ifngr1tm1Agt/J	Jackson Laboratory	RRID:IMSR_JAX:003,288
Mouse: B6.Cg-Rag2tm1.1Cgn TcrbLn5Sdou TcraLn3Sdou/J	Dougan et al.^57^	RRID:IMSR_JAX:030,958

Oligonucleotides		

GCGAGTCTCGAGATGTGTCCTCAGAAGCTAACC	IDT	XhoI_IL12_Fow
ATAGAAGCGGCCGCTCAGGCGGAGCTCAGATAG	IDT	NotI_IL12_Rev
CAGGCGCCCAATACGACCAAATC	Merkl et al.^77^	GAPDH fow DNA
TTCGACAGTCAGCCGCATCTTCTT	Merkl et al.^77^	GAPDH rev DNA
CAGGCGCCCAATACGACCAAATC	Merkl et al.^77^	ICP8 fow DNA
GAGACCGGGGTTGGGGAATGAATC	Merkl et al.^77^	ICP8 rev DNA
GTAACCCGTTGAACCCCATTCGT	IDT	m18S fow
CCATCCAATCGGTAGTAGCGAC	IDT	m18S rev
TGAAGCTTGACGCGGTACAT	IDT	mIfit2 fow
GCTGCCCTGAGGAGTGTATC	IDT	mIfit2 rev
ACCACAGCCCTCTCCATCAA	IDT	mIfnb1 fow
TTGAAGTCCGCCCTGTAGGT	IDT	mIfnb1 rev
AGCCGATGGGTTGTACCTTG	IDT	mTNF fow
ATAGCAAATCGGCTGACGGT	IDT	mTNF rev
CACTTCACAAGTCGGAGGCT	IDT	mIl6 fow
CTGCAAGTGCATCATCGTTGT	IDT	mIl6 rev
TGCAAGCTATGGCTCACTTCA	IDT	mIl1a fow
CTTCCCGTTGCTTGACGTTG	IDT	mIl1a rev
TCCTCCAAAACACTGCTGACA	IDT	mSting1 fow
GAGTCAAGCTCTGAAGGCCC	IDT	mSting1 rev
GGAAAGTCGTAAGGGGACCT	IDT	mCgas fow
TAGCTTGTCCGGTTCCTTCCT	IDT	mCgas rev
CGCTGCAACTGCATCCATATC	IDT	mCxcl10 fow
GATAGGCTCGCAGGGATGAT	IDT	mCxcl10 rev
GCAAACTTCCGTTGTGCCAT	IDT	mIrf1 fow
TCGGCTGGACTTGGACTTTC	IDT	mIrf1 rev
ACGCTGCCTATGATGTCTCG	IDT	mStat1 fow
AGAAAAGCGGCTGTACTGGT	IDT	mStat1 rev
GGTGTACGAACTTAGCCGGG	IDT	mIrf7 fow
AATGATCCTGGGGACACACC	IDT	mIrf7 rev

Recombinant DNA		

Tandem p40p35	Addgene	Addgene plasmid #108665
pSpCas9(BB)-2A-Puro (PX459) V2.0	Ran et al.^78^	Addgene plasmid #62988
lentiCRISPRv2	Sanjana et al.^79^	Addgene plasmid #52961
pVSV-G	Stewart et al.^80^	Addgene plasmid #8454
psPAX2	Addgene	Addgene plasmid #12260
hNectin-1	Nakashima et al.^45^	N/A
pd27B HSV-1 shuttle plasmid	Oh et al.^76^	N/A
pd27-IL12 HSV-1 shuttle plasmid	This paper	N/A
Infectious *d*106S DNA	Watanabe et al.^42^	N/A

Software and algorithms		

Fiji/ImageJ2	Schindelin et al.^81^	RRID:SCR_002285
cellSens Imaging software	Olympus	RRID:SCR_014551
FlowJo v10.8	TreeStar	RRID:SCR_008520
Prism v9	GraphPad	RRID:SCR_002798
Cellranger v6.1.0	10x Genomics	http://software.10xgenomics.com/; RRID:SCR_023221
R v4.2.2	R Project	https://www.r-project.org/; RRID:SCR_001905
RStudio v2022.12.0	Posit	https://posit.co/downloads/; RRID:SCR_000432
DropletUtils v1.10.3	Griffiths et al.^82^	Bioconductor
Seurat v4.0.2	Stuart et al.^83^	https://satijalab.org/seurat/
fgsea v.1.16.0	https://doi.org/10.1101/060012	Bioconductor
Molecular Signatures Database	Liberzon et al.^84^	https://www.gsea-msigdb.org/gsea/msigdb/index.jsp; RRID:SCR_016863
Salmon v1.8.0	Patro et al.^85^	https://combine-lab.github.io/salmon/
tximport v1.18.0	Soneson et al.^86^	Bioconductor
DESeq2 v1.30.1	Love et al.^87^	Bioconductor
Gencode v27	http://ftp.ebi.ac.uk/pub/databases/gencode/Gencode_mouse/	http://ftp.ebi.ac.uk/pub/databases/gencode/Gencode_mouse/
ggplot2 v3.3.6	Hadley Wickham	https://ggplot2.tidyverse.org/
cBioPortal	Cerami et al.^88^; Gao et al.^89^	https://www.cbioportal.org/; RRID:SCR_014555
survival v3.2-13	Terry M Therneau	https://CRAN.R-project.org/package=survival
survminer v0.4.9	Alboukadel Kassambara	https://cran.r-project.org/web/packages/survminer/index.html
Gene Ontology Resource	http://geneontology.org/	http://geneontology.org/; RRID:SCR_002811

## Highlights

- Model of cancer immune equilibrium using a non-replicating virus expressing IL-12
- IFNγ is critical for establishing and maintaining equilibrium
- Multiple mechanisms of T cell cytotoxicity are redundant for stable tumor control
- Tumors deficient for MHC class I or IFNγ sensing can be stably controlled
