# Supplementary material for: IFNγ is a central node of cancer immune equilibrium
Source: Cell Rep. Author manuscript; Available in PMC 2023 May 26. (PMC10214249; doi:10.1016/j.celrep.2023.112219)
Supplement: 1 [file NIHMS1887288-supplement-1.pdf]

Cell Reports, Volume *42*

Supplemental information

IFNγ is a central node of cancer immune equilibrium

Michael J. Walsh, Courtney T. Stump, Rakeeb Kureshi, Patrick Lenehan, Lestat R. Ali, Michael Dougan, David M. Knipe, and Stephanie K. Dougan

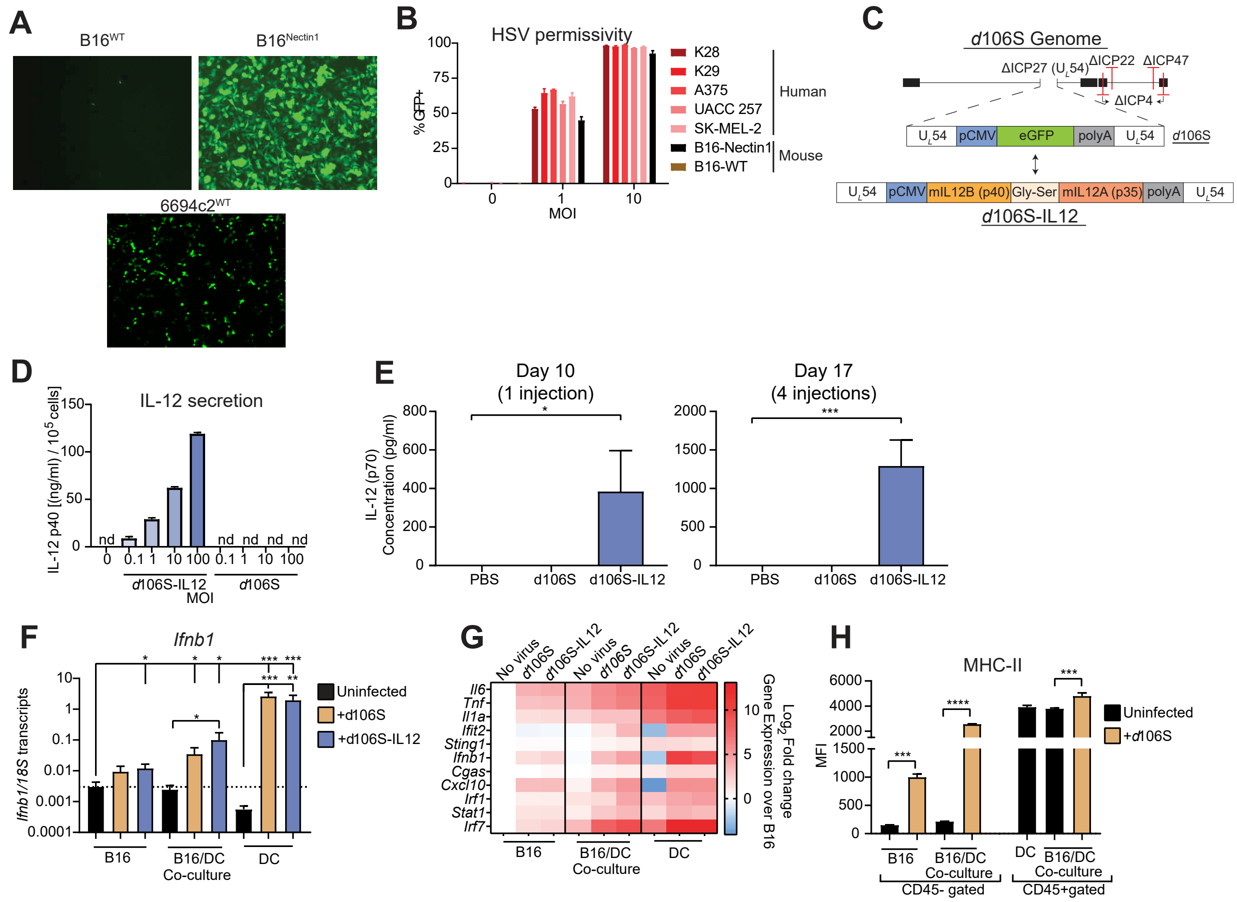

Figure S1: Generation of a replication-defective HSV-1 vector expressing IL-12 and inflammatory response by the vector. B16[WT] cells were transduced with a nectin-1 encoding lentivirus to generate B16[Nectin1] cells. (A) Fluorescence micrograph of B16[WT], B16[Nectin1] murine melanoma cells or Wild-type 6694c2 pancreatic cancer cells infected with *d*106S-GFP at MOI 10 for 24 hours. (B) A panel of five human melanoma cells or murine B16 (Nectin1 or WT) melanoma cells were infected at multiplicity of infection (MOI) 0, 1, or 10 with *d*106S (GFP). Percent GFP-positive cells were determined at 24 hours post infection (hpi) by flow cytometry. (C) Map of the *d*106S genome, which has deletions of 4 genes, rendering it replication defective. ICP27 (U_L54) has been replaced with CMV-promoter-driven GFP expression. Using homologous recombination, an IL-12 fusion protein (p40-GlySer-p35) replaced the GFP expression to generate *d*106S-IL12. B16[Nectin1] cells infected at varying MOIs with *d*106S or *d*106S-IL12. (D) Supernatants of infected B16[Nectin1] cells were collected 24 hpi and analyzed for IL-12 (p40) by ELISA. (E) B16[Nectin1] tumors were injected at day seven once or four times every three days and collected on day 10 or day 17, respectively for cytokine multiplex. (F and G) B16[Nectin1] cells were infected at MOI 10 with *d*106S or mock infected. Infected and uninfected tumor cells were washed and either monocultured or co-cultured with bone-marrow derived DCs. DCs were also incubated with virus at MOI 10. Total RNA was harvested 24 hpi and mRNA expression analyzed against a panel of inflammatory genes. mRNA levels were normalized to 18S rRNA. Dashed line indicates gene expression of uninfected B16. (H) B16[Nectin1] cells were infected with *d*106S (MOI 10) and mono- or co-cultured with DCs for 24 hours and I-A/I-E (MHC-II) expression analyzed by flow cytometry, gating on either CD45- B16[Nectin1] or CD45+ DCs (MFI; mean fluorescence intensity). Values are mean ± SEM, N = 3-4. Groups were compared with a one-way ANOVA and Sidak's or Dunnett's multiple comparisons test.

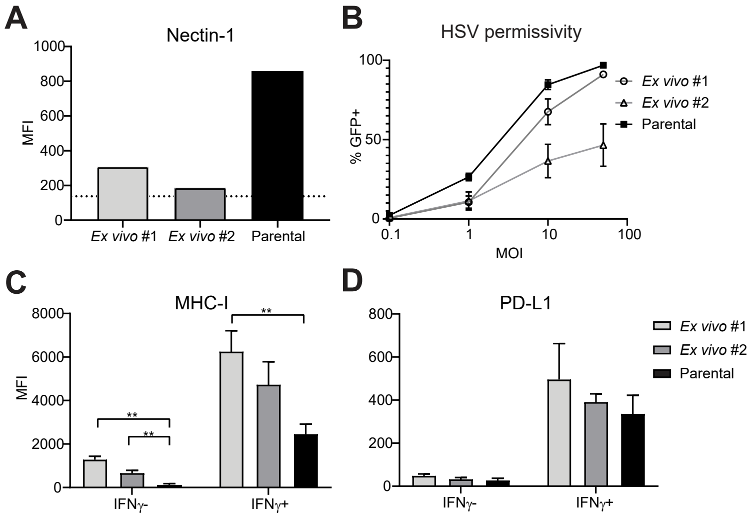

Figure S2: Characeristics of *ex vivo* tumors following *d*106S-IL12 (related to Figure 2). *Ex vivo* cells were derived from Figure 2B. (A) Nectin-1 levels of *ex vivo* and parental $B16^{Nectin1}$ analyzed by flow cytometry. Representative of several independent experiments. (B) *Ex vivo* tumor cultures or parental $B16^{Nectin1}$ cells were infected with *d*106S-GFP at various MOIs and analyzed by flow cytometry at 24 hpi. (C and D) *Ex vivo* and parental $B16^{Nectin1}$ cells were left untreated or stimulated with 20ng/mL of IFNγ for 24 hours before analyzing $H2\text{-}K^b/D^b$ (MHC-I) and PD-L1 levels by flow cytometry. Values are mean ± SEM, N = 3-4. Groups were compared with a two-way ANOVA and mixed-effects multiple comparisons test

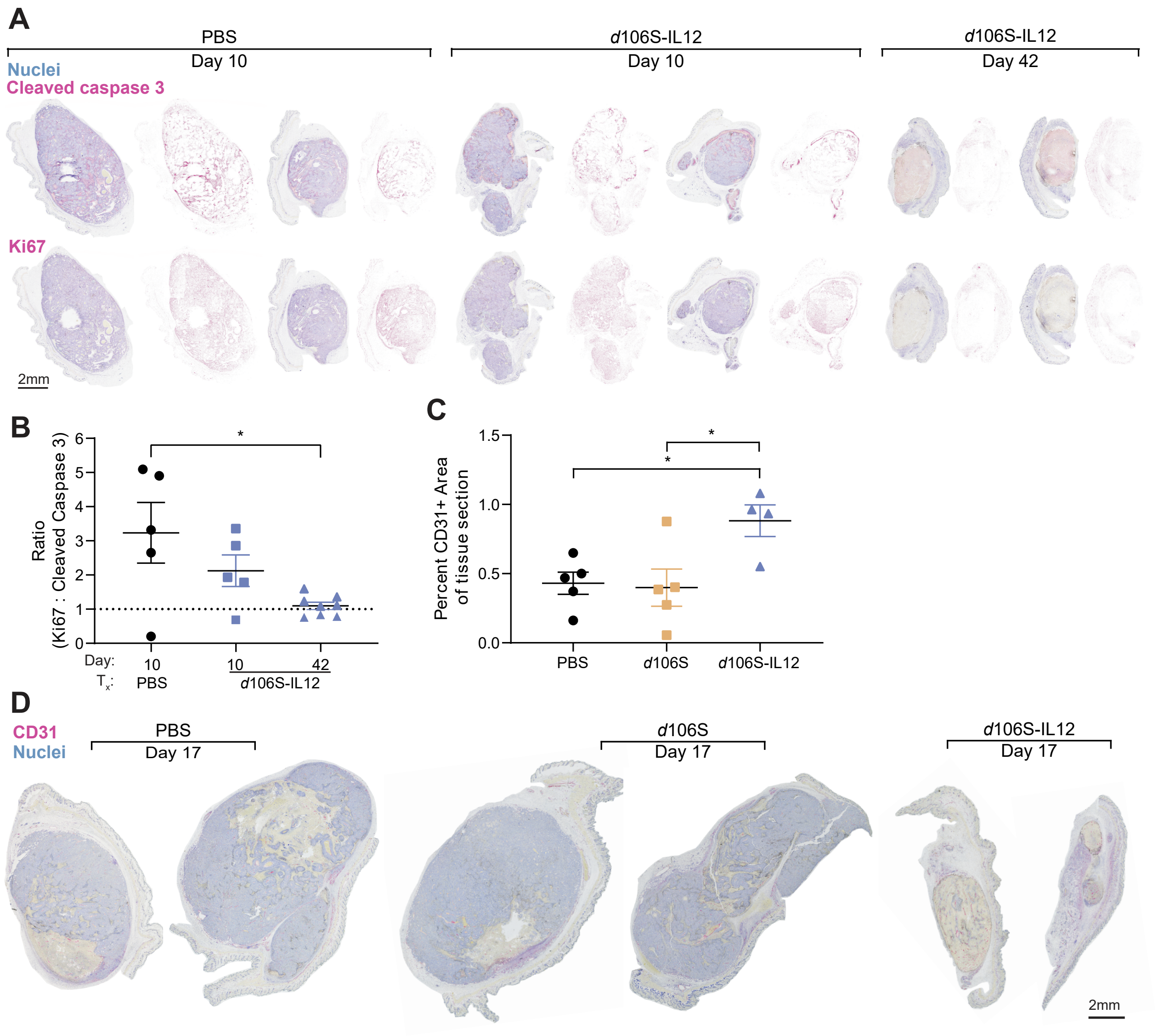

Figure S3: Tumor vasculature, apoptosis, and proliferation during *d*106S-IL12 therapy (related to Figure 2) Fixed, paraffin embedded tumors from Figure 2E-2H were stained and adjacent sections were stained separately for cleaved caspase 3 or Ki67. (A) Representative full tissue sections including deconvoluted, stain-only images displayed next to original sections. (B) ImageJ was used to quantify percent area of cleaved caspase 3 and Ki67, from which a ratio was calculated. (C, D) Mice with B16^{Nectin1} tumors were treated four times total and sacrificed on day 17 (N = 5 PBS/*d*106S, N = 4 *d*106S-IL12). Tumors and surrounding tissue were fixed, paraffin embedded and tissue sections were stained for CD31. Quantification of percent CD31^{+} area per full tissue section using ImageJ. (D) Representative full tissue sections from (C). Values are mean ± SEM.Groups were compared with a one-way ANOVA and Tukey's multiple comparisons test.

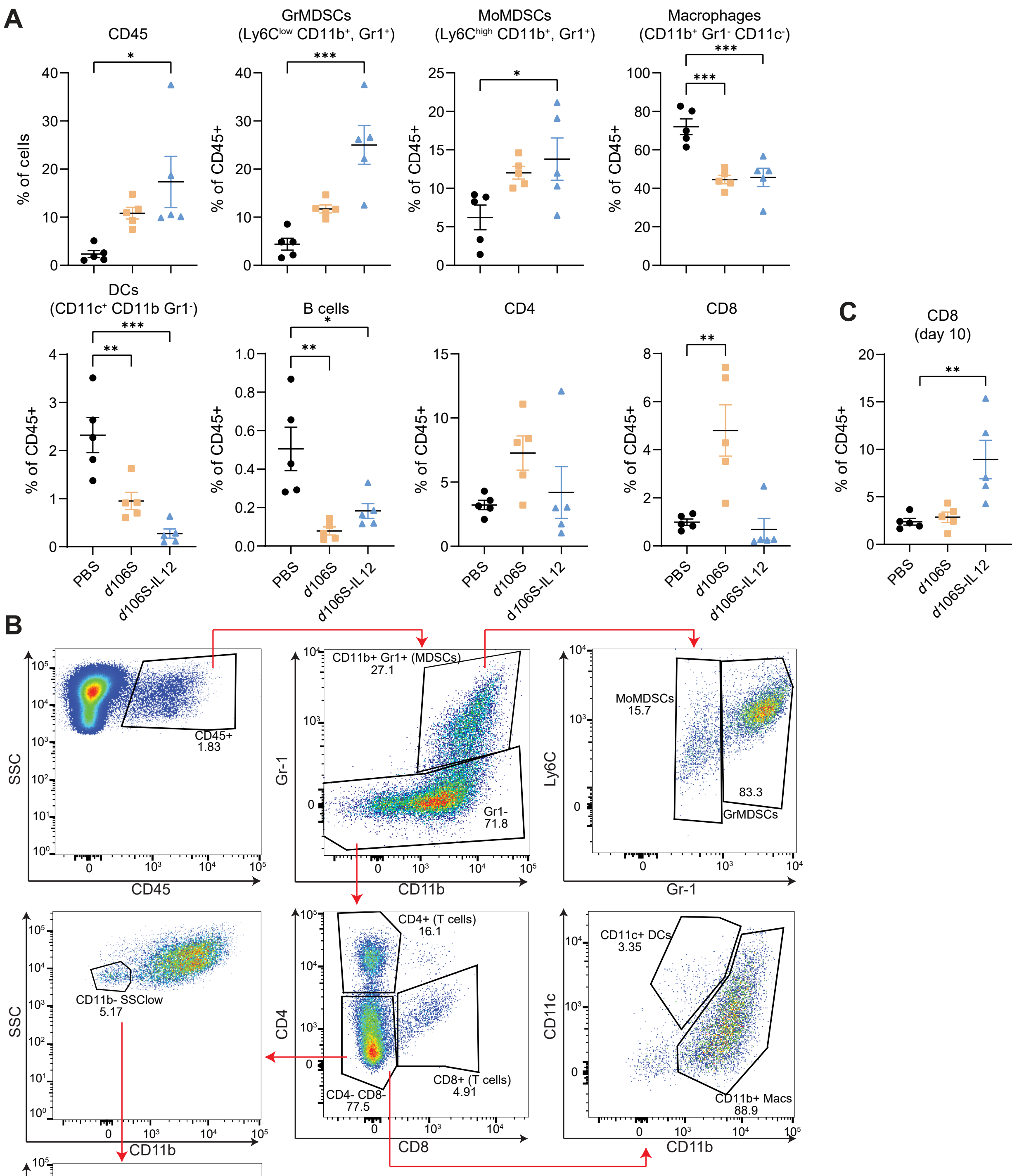

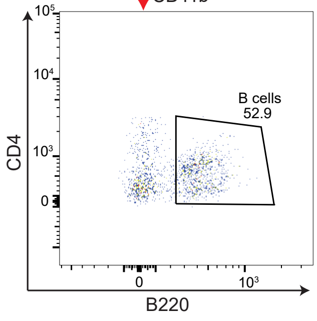

Figure S4: Infiltrating cells in tumors treated with *d*106S-IL12 (related to Figure 3). (A) Tumors processed for single-cell RNA-sequencing in Figure 3 were also analyzed by flow cytometry. (B) Representative gating strategy shown. (C) Mice bearing B16[Nectin1] tumors were treated just once intratumorally at day seven and $CD8^+$ T cells were measured by flow cytometry at day 10. Values are mean ± SEM. Groups were compared with a one-way ANOVA and Dunnett's multiple comparisons test.

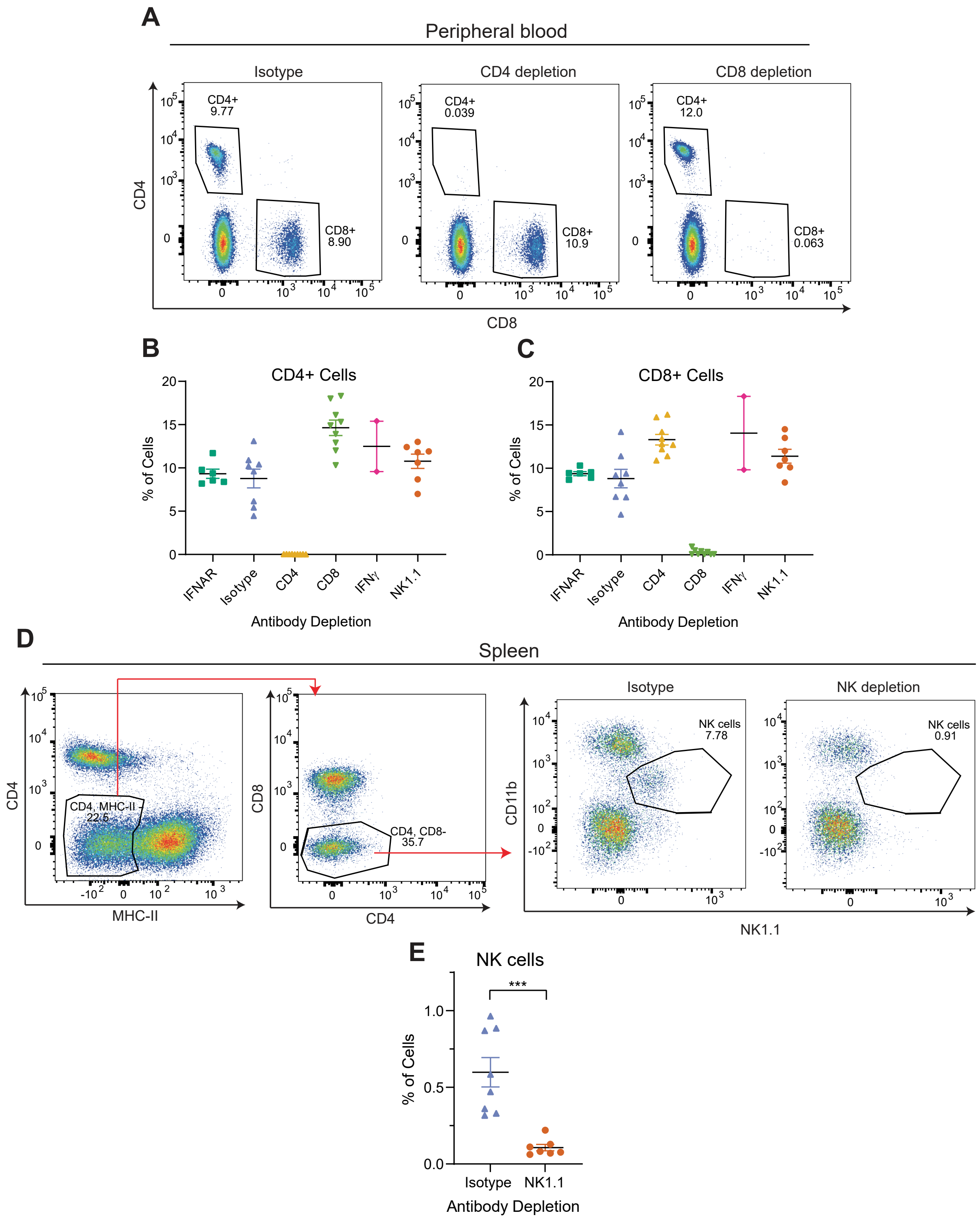

Figure S5: Antibodies successfully deplete target cell types (related to Figure 4). Remaining mice from Figure 2D were bled retrorbitally at day 62 and peripheral blood stained to confirm the depletion of (A, B) CD4+ T cells and (A, C) CD8+ T cells by flow cytometry. (D, E) At day 63, these mice were sacrificed and their spleens stained to confirm depletion of NK cells by flow cytometry. Representative gating shown. Values are mean ± SEM. NK cells were compared using an unpaired t-test.

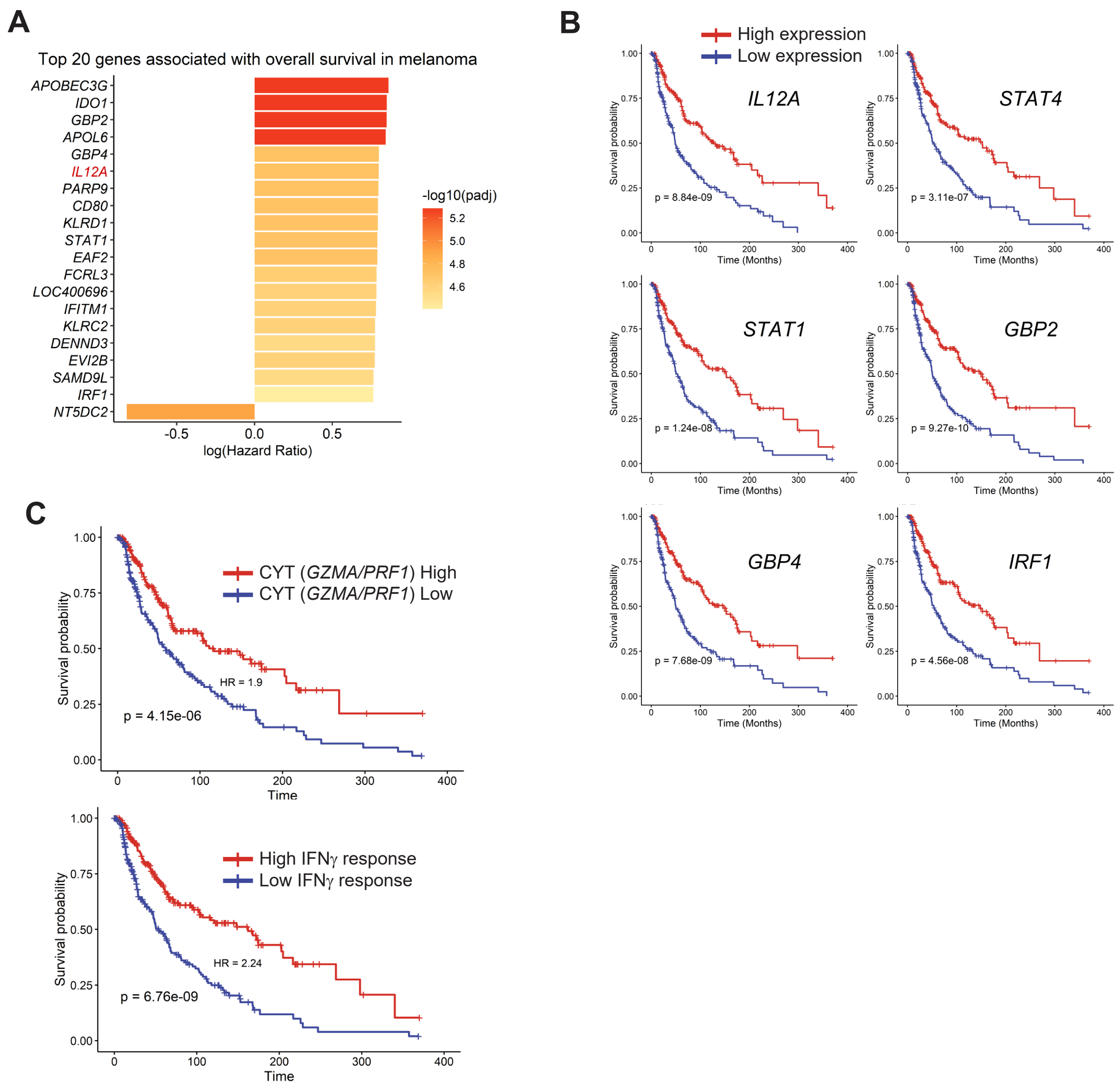

Figure S6: Increased IFNγ response is associated with better survival outcome in human melanoma. Transcriptomic data from The Cancer Genome Atlas (TCGA) for melanoma patients was analyzed across all genes; patients were stratified into expression above/below the median for each gene and differences in overall survival between high/low expression were compared. P-values were adjusted for false-discovery using Benjamini-Hochberg correction. (A) Top 20 most significant genes in melanoma associated with increased, positive log(Hazard Ratio), or decreased survival, negative log(Hazard Ratio). (B) Select IL-12 and interferon-stimulated genes associated with increased survival in melanoma. (C) Melanoma survival with high/low CYT (geometric mean of *GZMA* and *PRF1* genes) as defined by Rooney *et al.*, 2015. Melanoma survival with high/low IFNγ-response (scaled and summed IFNγ-stimulated gene expression). HR = Hazard ratio. Survival compared by log-rank test. P-values shown on Kaplan-Meier curves are unadjusted.

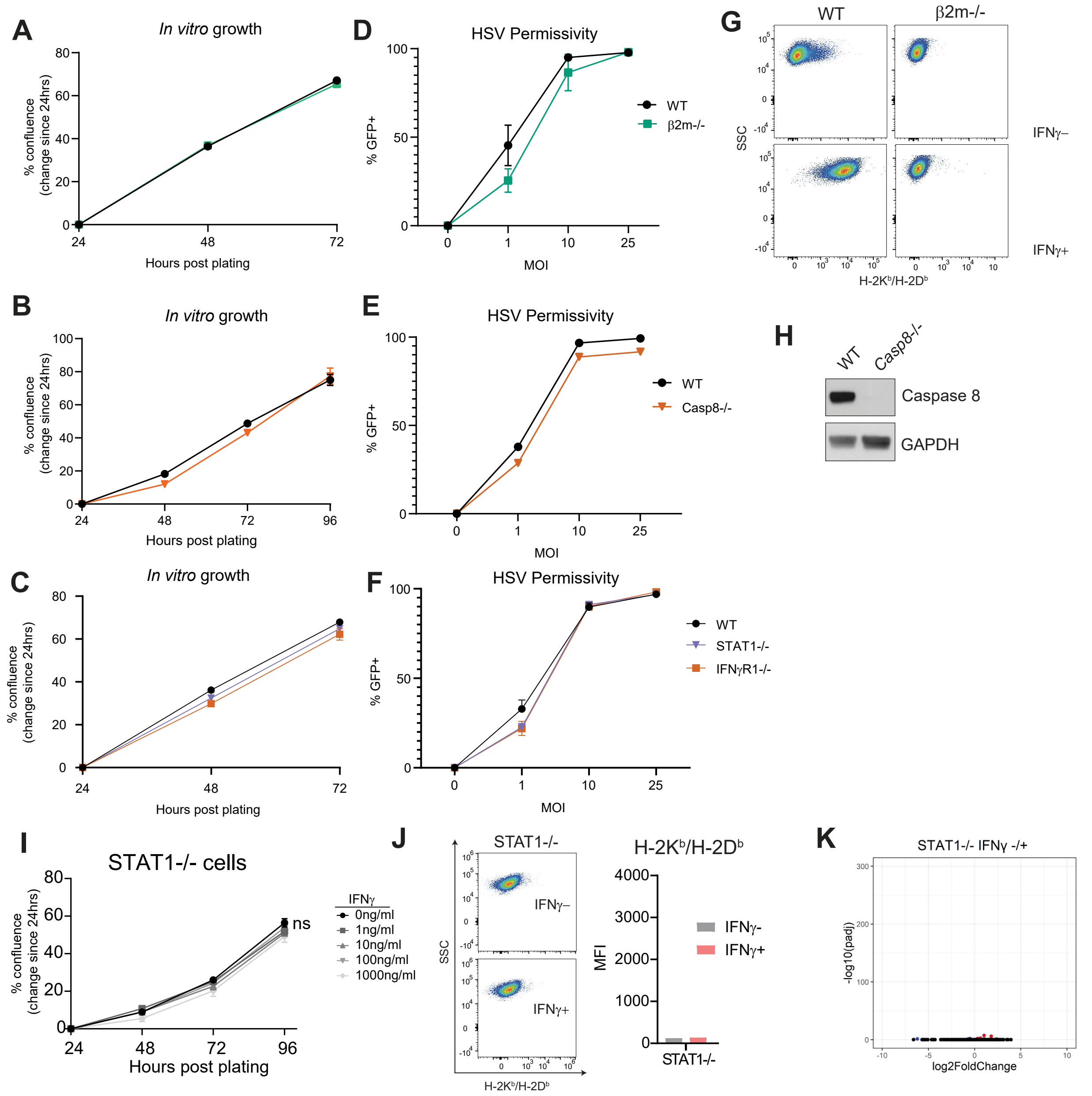

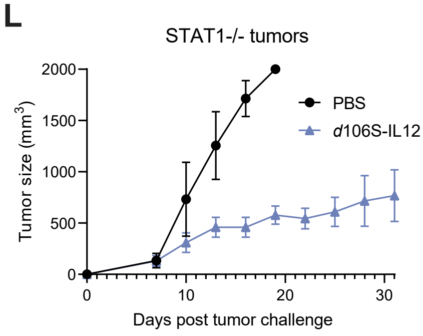

Figure S7: B16 knockout characterizations (related to Figures 5 and 6). CRISPR-Cas9 gene editing was used to generate β2m, Caspase 8, STAT1 and IFNγR1 knockout B16 cell lines. These cell lines were transduced with a nectin-1 encoding lentivirus to generate cell lines permissive to HSV entry. These B16 knockout, nectin-1+ cells were compared to the original wild-type, nectin-1+ cells used in all prior experiments. (A-C) Cells were plated and confluence determined every 24 hours by Celigo image cytometer. (D-F) Cells were infected at an MOI 0, 1, 10, and 25 with *d*106S-GFP and percent GFP-positive cells were determined at 24 hours post infection by flow cytometry. (G) Wild-type, or β2m-/- cells were cultured *in vitro* with 50ng/ml IFNγ and H2-K^b/D^b (MHC-I) expression determined by flow cytometry 24 hours later. (H) Western blot of Caspase 8-/- cells one week following Cas9 editing. (I) STAT1-/- growth curve associated with Figure 6A. (J) STAT1-/- MHC-I staining associated with Figure 6B. (K) RNA-sequencing of STAT1-/- cells associated with Figure 6C. (L) STAT1-/- tumor growth curve with *d*106S-IL12 treament; corresponding wild-type (and β2m-/-) tumors depicted in Figure 5B.
