# Supplementary material for: IFNγ is a central node of cancer immune equilibrium
Source: Cell Rep. Author manuscript; Available in PMC 2023 May 26. (PMC10214249; doi:10.1016/j.celrep.2023.112219)
Supplement: 2 [file NIHMS1887288-supplement-2.pdf]

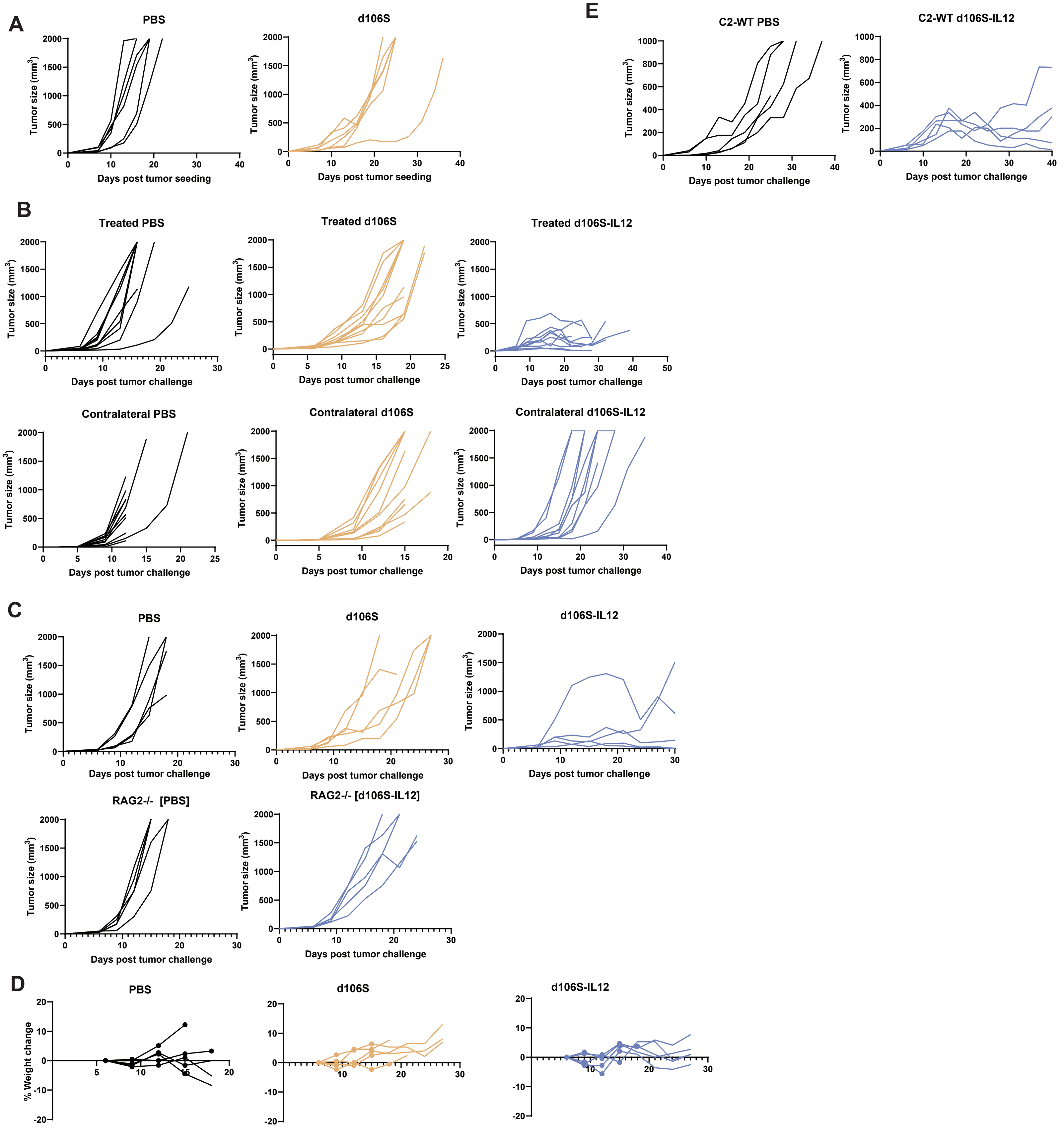

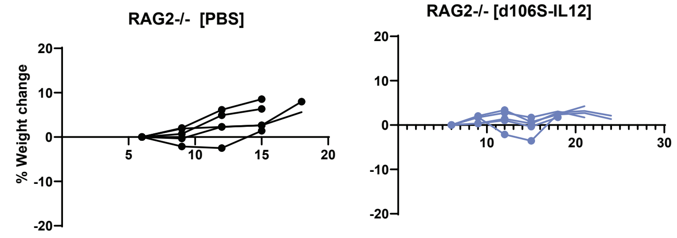

Data S1 Individual growth curves and weight curves of mice(related ot Figure 1).

A) growth curves related to Figure 1A
B) growth curves related to Figures 1B/C
C) growth curves related to Figures 1D/E
D) weight change related to Figures 1D/E
E) growth curves related to Figure 1H

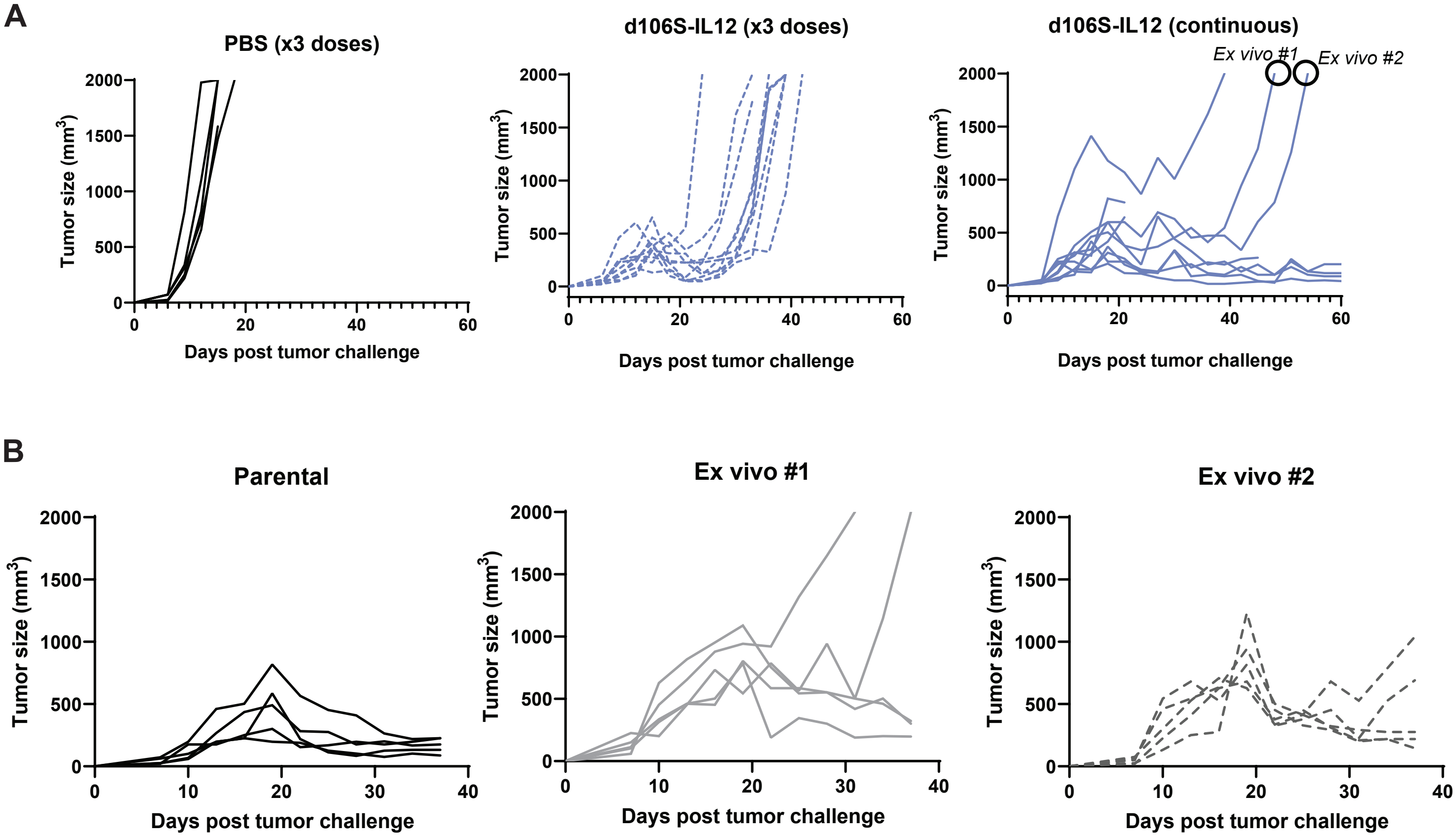

Data S2 Individual growth curves of mice(related ot Figure 2).

A) growth curves related to Figure 2A

B) growth curves related to Figure 2D

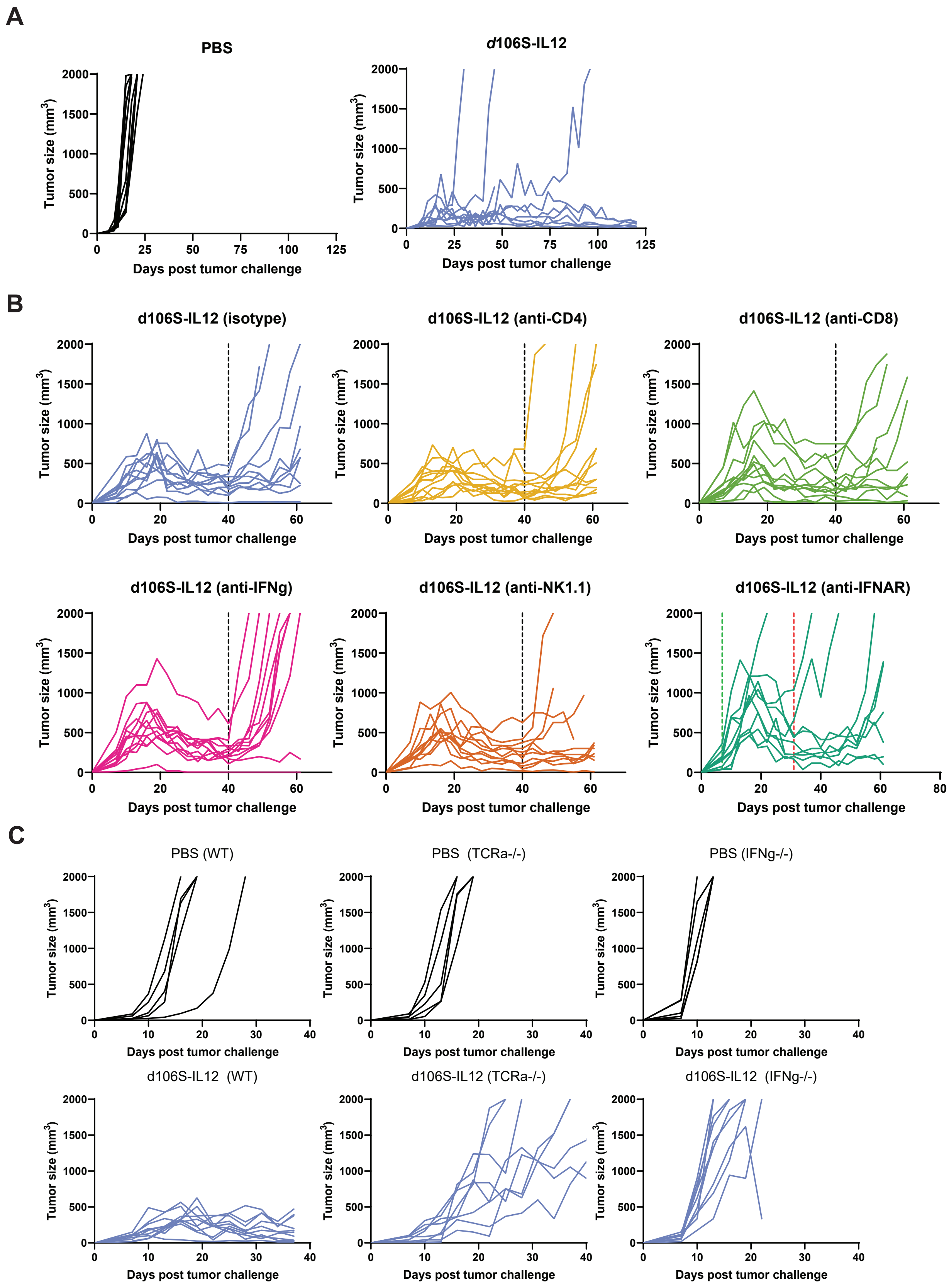

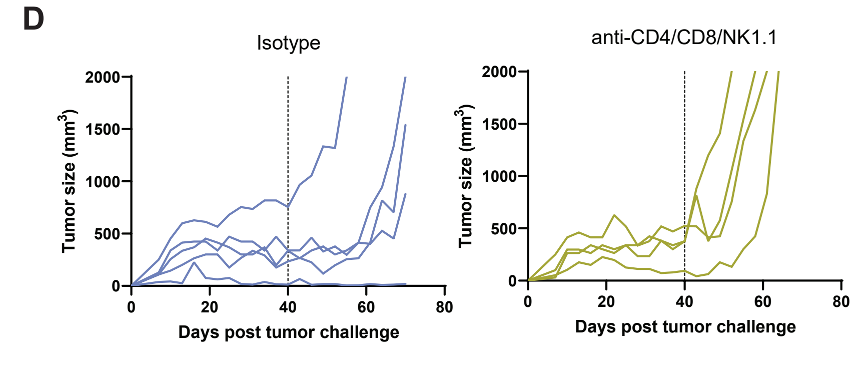

Data S3 Individual growth curves of mice (related to Figure 4).

A) growth curves related to Figures 4A/B

B) growth curves related to Figure 4C

C) growth curves related to Figure 4D

D) growth curves related to Figure 4E

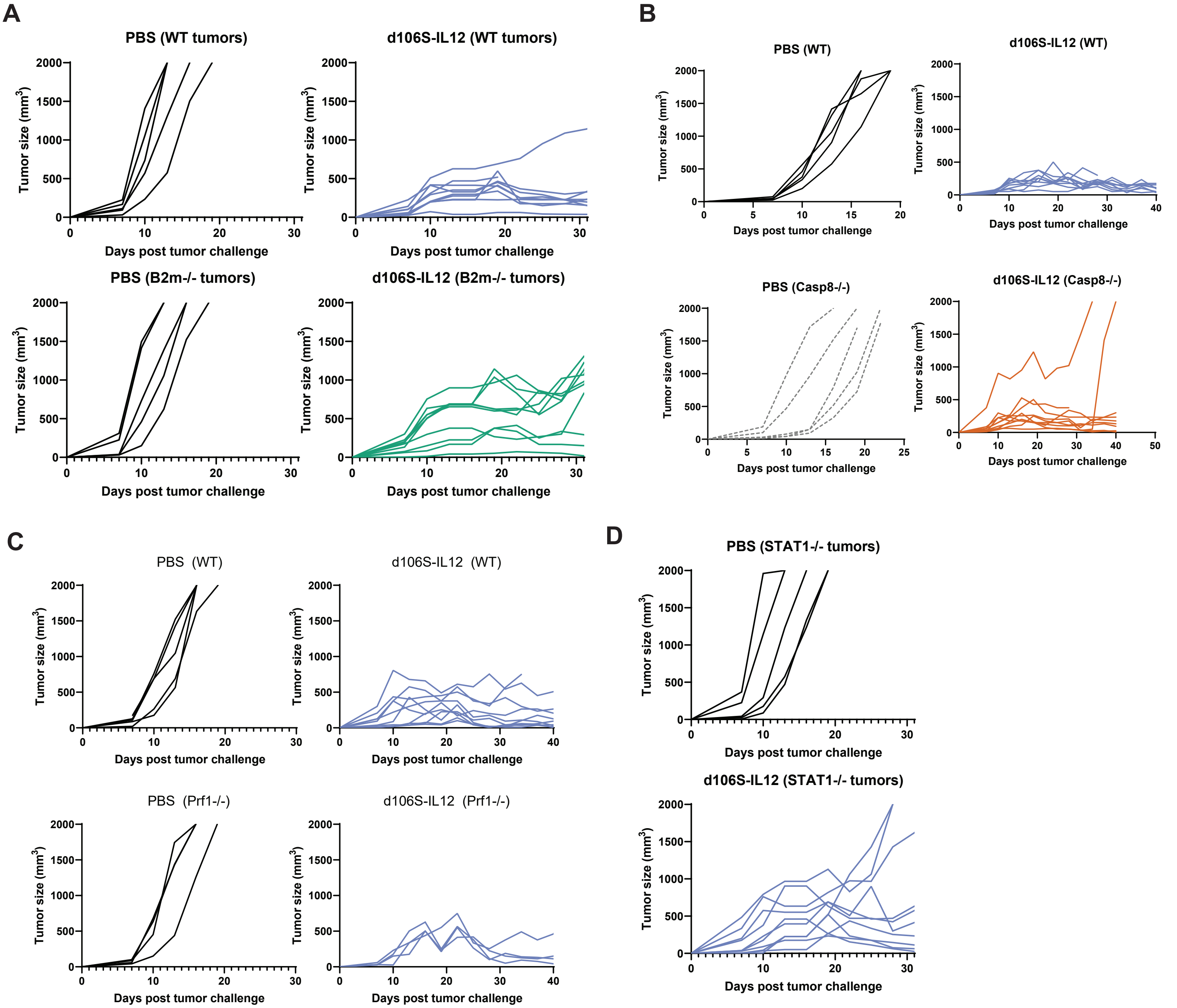

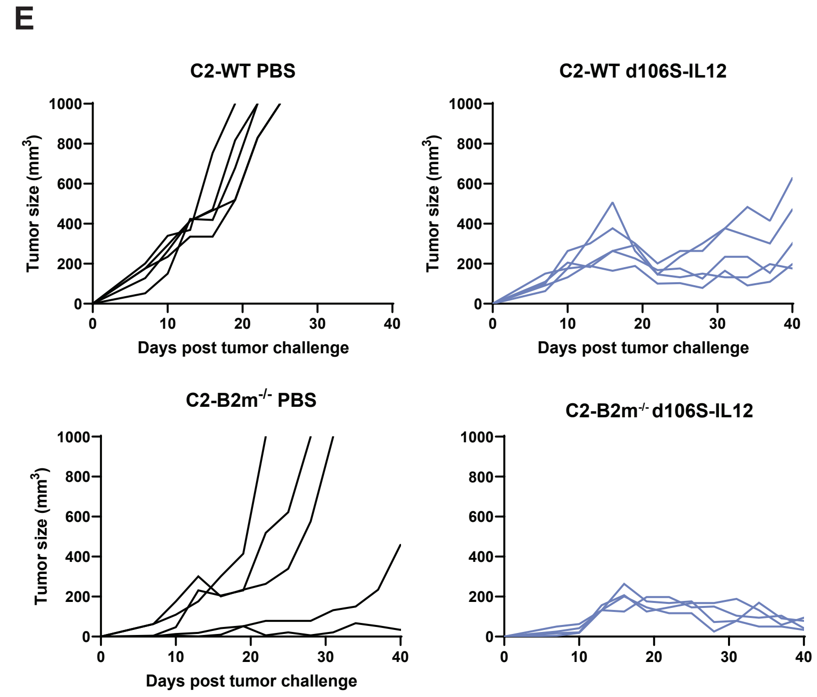

Data S4 Individual growth curves of mice (related to Figures 5, S7).

A) growth curves related to Figures 5B
B) growth curves related to Figure 5C
C) growth curves related to Figure 5D
D) growth curves related to Figure S7L
E) growth curves related to Figure 5E

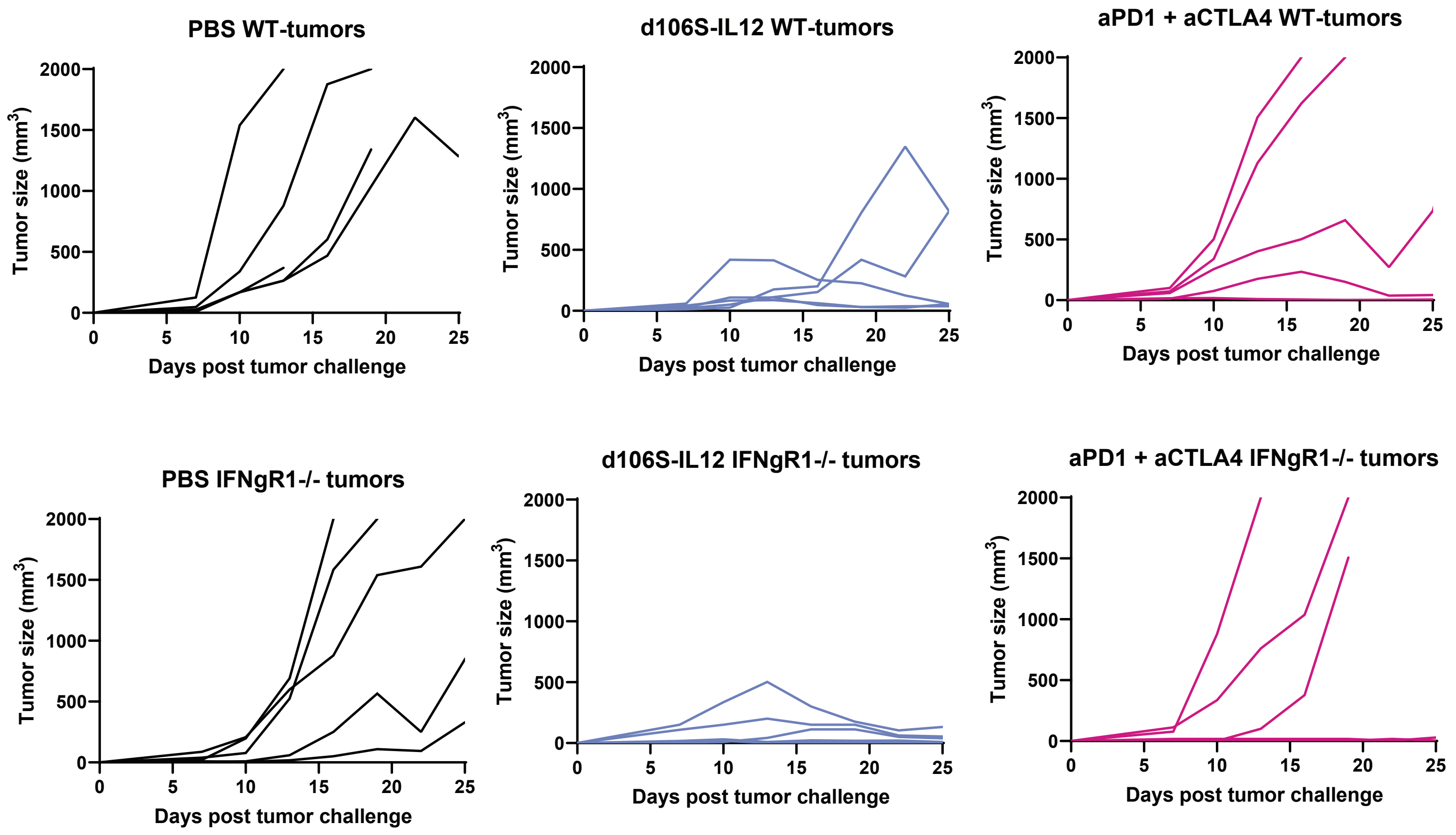

Data S5 Individual growth curves of mice (related to Figure 6).
Growth curves related to Figure 6D

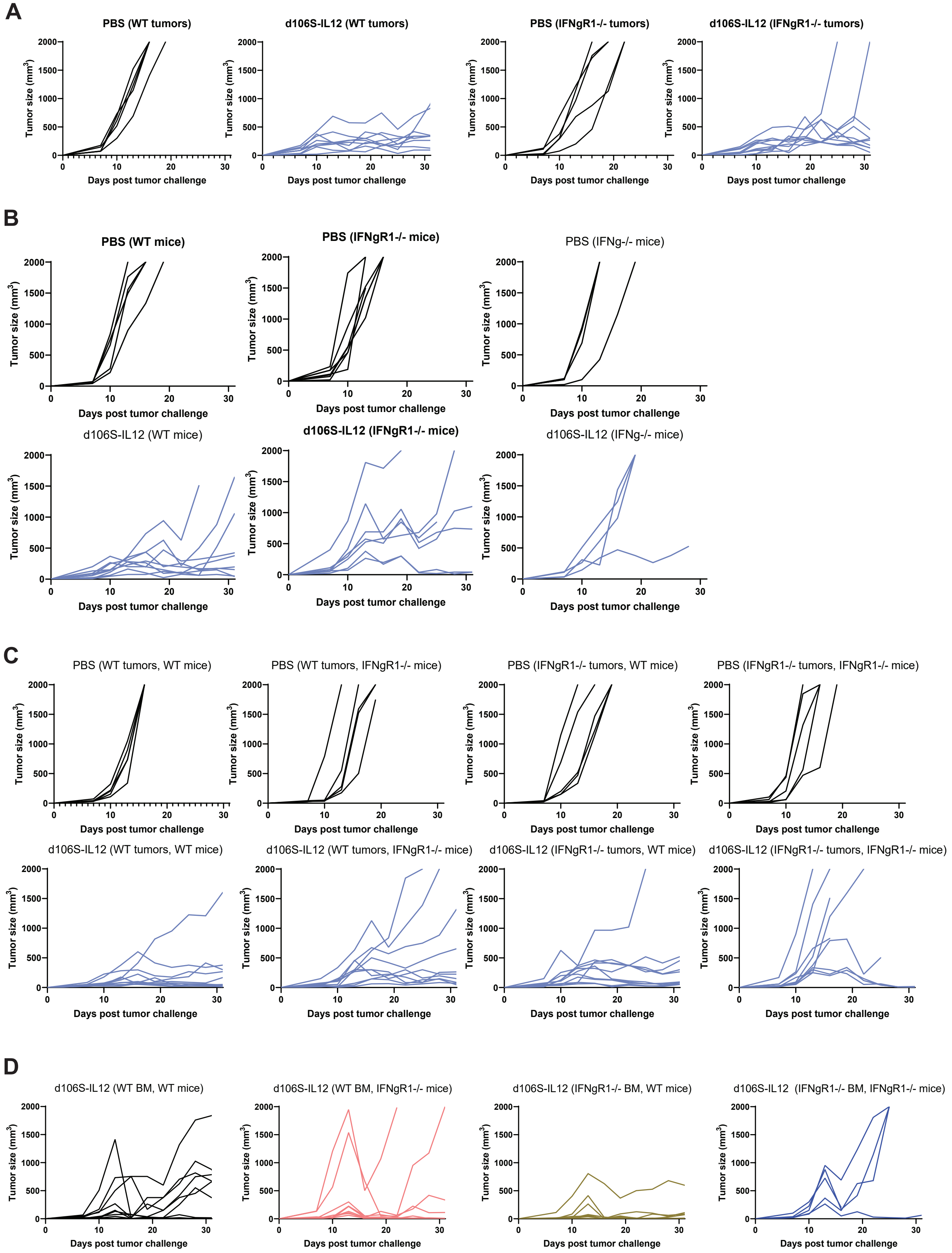

Data S6 Individual growth curves of mice (related to Figure 7).

A) growth curves related to Figures 7A
B) growth curves related to Figure 7B
C) growth curves related to Figure 7C
D) growth curves related to Figure 7D
